# An engineered bacterial therapeutic lowers urinary oxalate in preclinical models and *in silico* simulations of enteric hyperoxaluria

**DOI:** 10.15252/msb.202110539

**Published:** 2022-03-07

**Authors:** David Lubkowicz, Nicholas G Horvath, Michael J James, Pasquale Cantarella, Lauren Renaud, Christopher G Bergeron, Ron B Shmueli, Cami Anderson, Jian‐Rong Gao, Caroline B Kurtz, Mylene Perreault, Mark R Charbonneau, Vincent M Isabella, David L Hava

**Affiliations:** ^1^ Synlogic Therapeutics Cambridge MA USA

**Keywords:** engineered bacteria, enteric hyperoxaluria, *in silico* modeling, oxalate, synthetic biology, Biotechnology & Synthetic Biology, Pharmacology & Drug Discovery

## Abstract

Enteric hyperoxaluria (EH) is a metabolic disease caused by excessive absorption of dietary oxalate leading to the formation of chronic kidney stones and kidney failure. There are no approved pharmaceutical treatments for EH. SYNB8802 is an engineered bacterial therapeutic designed to consume oxalate in the gut and lower urinary oxalate as a potential treatment for EH. Oral administration of SYNB8802 leads to significantly decreased urinary oxalate excretion in healthy mice and non‐human primates, demonstrating the strain's ability to consume oxalate *in vivo*. A mathematical modeling framework was constructed that combines *in vitro* and *in vivo* preclinical data to predict the effects of SYNB8802 administration on urinary oxalate excretion in humans. Simulations of SYNB8802 administration predict a clinically meaningful lowering of urinary oxalate excretion in healthy volunteers and EH patients. Together, these findings suggest that SYNB8802 is a promising treatment for EH.

## Graphical abstract

SYNB8802 is an engineered probiotic designed to metabolize oxalate in patients with enteric hyperoxaluria. The strain shows significant oxalate degradation *in vitro* and *in vivo*, and *in silico* modeling predicts that it can lower urinary oxalate in patients.

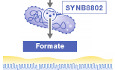

## Introduction

Oxalate is an end‐product of human metabolism and is present in a variety of common foods including green vegetables, nuts, grains, fruits, and chocolate (Attalla *et al,* 2014). A fraction of dietary oxalate is absorbed by the gastrointestinal (GI) tract, and systemic oxalate is excreted by the kidneys as urinary oxalate (UOx) (Mitchell *et al*, 2019). The contribution of endogenous oxalate production and dietary oxalate absorption to UOx excretion are approximately equal in healthy individuals (Holmes *et al*, 2001), but an elevation of UOx can arise from increased GI oxalate absorption (Nazzal *et al*, 2015). Enteric hyperoxaluria (EH) does not have a clear genetic link, but is commonly observed in patients with underlying GI disease associated with fat malabsorption, such as inflammatory bowel disease, as increased luminal free fatty acids increase oxalate solubility and absorption (Dobbins & Binder, 1976; Hylander *et al*, 1978).

Chronic EH is associated with recurrent kidney stones, nephrocalcinosis, and chronic kidney disease (Nazzal *et al*, 2015). Progressive renal damage from increased stone frequency can lead to kidney failure and the need for kidney transplantation (Asplin & Coe, 2007). Renal failure may recur and necessitate additional transplantation if the underlying disease is not addressed. In addition, untreated EH can progress to systemic oxalosis, a condition in which oxalate accumulates in joints, bones, eyes, heart, and other organs (Hueppelshaeuser *et al*, 2012). EH patients with UOx levels consistently above the upper limit of normal have a higher risk of kidney stone events (D'Costa *et al*, 2021). Lowering UOx is predicted to decrease the odds of a patient developing a kidney stone. An accelerated failure time model, based on a recent epidemiological study in 279 patients with EH, predicted that a 20% decrease in UOx levels reduces the risk of a kidney stone event by 25% (D'Costa *et al*, 2021). There are currently no approved pharmacological therapies for EH, and disease management aims to decrease the risk of recurrent kidney stones by limiting dietary oxalate and fat, increasing dietary calcium intake, and maintaining adequate fluid intake (Pearle *et al*, 1999; Siener *et al*, 2021). However, the efficacy of dietary modifications alone is limited, especially in patients with severe hyperoxaluria (Schwen *et al*, 2013), and lifelong adherence to a low oxalate diet is challenging (Attalla *et al*, 2014). There is a clear unmet need for effective therapies that reduce oxalate levels in EH patients and their associated complications.

Certain members of the human gut microbiota degrade oxalate and may modulate intestinal oxalate secretion (Hatch & Freel, 2013; Knight *et al*, 2013). *Oxalobacter formigenes* (OF) utilizes oxalate as an energy source and has been evaluated as a potential therapeutic for hyperoxaluria (Jairath *et al*, 2015; Milliner *et al*, 2018). However, as a strict anaerobe, manufacturing challenges may exist (Charbonneau *et al*, 2020). OF colonizes humans and a colonizing therapeutic must compete with natural members of the host microbiota for an ecological niche (Bauer *et al*, 2018). Controlling colonization of bacterial therapeutics may be complex and the withdrawal of such therapies may require treatment with antibiotics to eliminate the colonizing strain. In contrast, *Escherichia coli* Nissle 1917 (EcN) is a non‐colonizing, probiotic strain (Kurtz *et al*, 2018), providing a flexible platform for the development of engineered bacterial therapeutics (synthetic biotics). EcN strains that consume toxic metabolites within the GI tract have been developed (Isabella *et al*, 2018) and are safe and well‐tolerated in healthy volunteers (Kurtz *et al*, 2019; Puurunen *et al*, 2021). The non‐colonizing nature of EcN enables predictable pharmacodynamics, reduced risk of genetic drift *in vivo*, limited interactions with host microbiota and the complete clearance after dosing is stopped (Charbonneau *et al*, 2020).

Moreover, mathematical modeling is a useful tool to translate preclinical evidence of drug action to clinical benefit and has been proven successful in the acceleration of drug development across a wide assortment of therapeutic indications (Milligan *et al*, 2013). The application of mechanistic modeling frameworks to live bacterial therapeutics combining preclinical data of strain activity with prior knowledge of human GI physiology enables the prediction of effects on biomarkers of disease and increased confidence regarding the translational potential of synthetic biotics in patients (Charbonneau *et al*, 2021; Nelson *et al*, 2021).

In this article, we describe the construction of a synthetic biotic, designated SYNB8802, that metabolizes oxalate within the GI tract. SYNB8802 degrades oxalate *in vitro* and reduces UOx excretion in mouse and non‐human primate models of hyperoxaluria. In addition, a mathematical model of SYNB8802 activity was developed to estimate the strain's impact on UOx excretion in healthy subjects and EH patients. This computational framework predicts a dose‐dependent lowering of UOx levels with daily oral administration of SYNB8802. Taken together, these data suggest that SYNB8802 has the potential to consume oxalate within the GI tract of EH patients and is a promising treatment for EH.

## Results

### SYNB8802 degrades oxalate *in vitro*

**Figure EV1:**
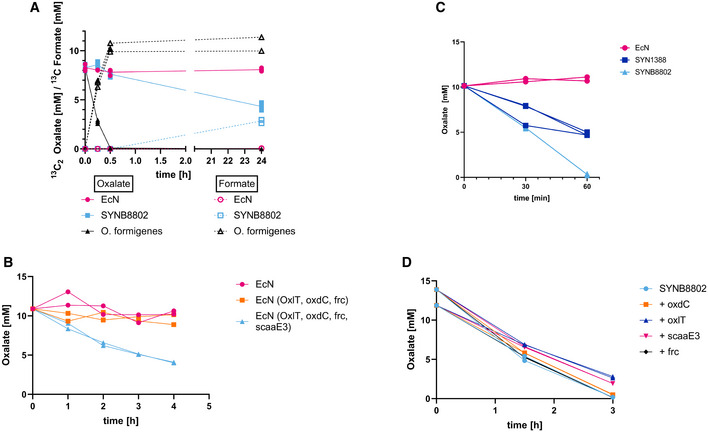
SYNB8802 *in vitro* evaluation SYNB8802 vs. *Oxalobacter formigenes*. Left *y*‐axis: ^13^C_2_‐oxalate in mM, ^13^C‐formate in mM, *x*‐axis: time in hours. *Oxalobacter formigenes* (black, triangle, solid line: oxalate, dotted line: formate). SYNB8802 (light blue, square, solid line: oxalate, dotted line: formate). The control EcN (pink, circle, solid line: oxalate, dotted line: formate). Biological triplicates were run and plotted separately.Scaae3 is needed for oxalate degradation in EcN. *Y*‐axis shows oxalate in mM. *X*‐axis: time in hours. Wild type EcN (pink, circle). EcN expressing the OF genes *oxdC, oxlT*, and *frc* on p15a (orange, square). EcN expressing the OF genes including *scaaE3* on p15a (light blue, triangle). Biological duplicates were run and plotted separately.SYNB8802 vs. SYN1388. *Y*‐axis shows oxalate in mM. *X*‐axis: time in minutes. Wild type EcN (pink, circle). Prototype strain SYN1388 (blue, square). SYNB8802 (light blue, triangle). Biological duplicates were run and plotted separately.Overexpression of individual pathway components. *Y*‐axis shows oxalate in mM. *X*‐axis: time in hours. SYNB8802 (blue, circle). SYNB8802, with added plasmid‐based expression of *oxdC* (orange, square). SYNB8802, with added plasmid‐based expression of *oxlT* (blue, triangle). SYNB8802, with added plasmid‐based expression of *scaaE3* (pink, triangle). SYNB8802, with added plasmid‐based expression of *frc* (black, diamond). Biological duplicates were run and plotted separately.

The role of OF in human oxalate metabolism has been extensively characterized (Liu *et al*, 2021). OF expresses an oxalate degradation pathway comprised of genes encoding the oxalate/formate antiporter (OxlT), oxalyl‐CoA decarboxylase (OxdC), and formyl‐CoA transferase (Frc). OxlT transports oxalate into the cytoplasm in exchange for formate. Frc then facilitates the exchange of CoA from formyl‐CoA to oxalate, yielding oxalyl‐CoA and formate. OxdC decarboxylates oxalyl‐CoA to yield formyl‐CoA and CO_2_, regenerating the substrates necessary for this cyclic reaction to proceed (Knight *et al*, 2013). The ability of *O*. *formigenes* to degrade oxalate in minimal media was evaluated using ^13^C_2_‐labeled oxalate. OF completely degraded 10 mM ^13^C_2_‐labeled oxalate to ^13^C‐formate within 30 min (Fig EV1A). We reasoned that engineering this OF pathway in EcN could endow the strain with oxalate consumption ability. However, no oxalate consumption was observed in EcN following cloning and expression of *oxlT, oxdC*, and *frc* (Fig EV1B), demonstrating that additional enzymatic activities are required. An extensive literature search did not reveal any described *E. coli* pathways that enable the generation of either oxalyl‐CoA or formyl‐CoA from oxalate or formate, respectively. Hence, we hypothesized that the missing function of the engineered EcN strain was the activation of oxalate or formate to their high energy ‐CoA intermediates, a requirement for the key decarboxylation step of the pathway to occur. To resolve this issue, an oxalyl‐CoA synthetase gene (*scaaE3*) from *Saccharomyces cerevisiae* (Foster & Nakata, 2014) was co‐expressed in EcN with *oxlT*, *oxdC*, and *frc*. Inclusion of *scaaE3* conferred the ability of the strain to significantly reduce oxalate levels *in vitro* (Fig EV1B).

**Figure 1:**
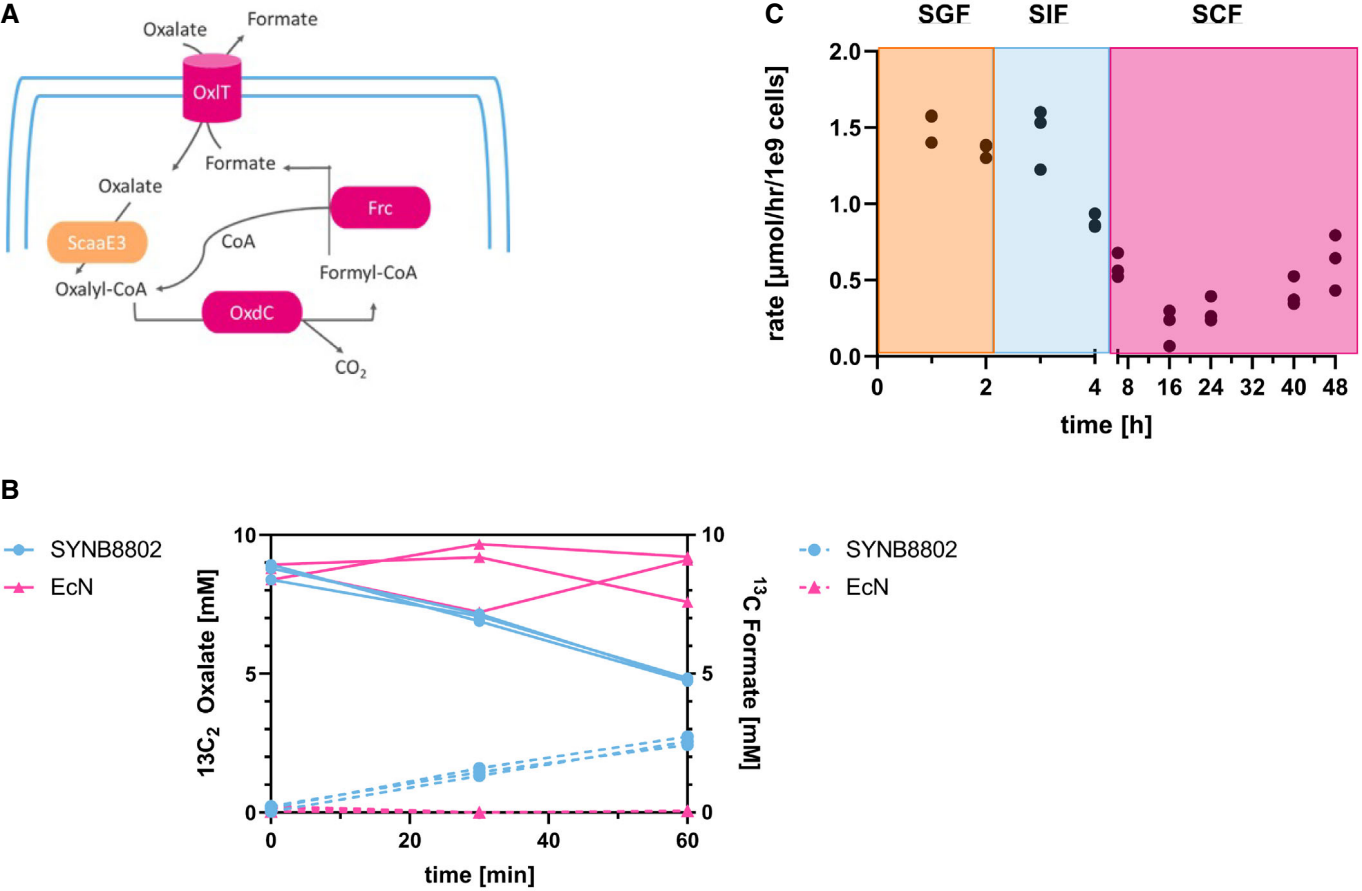
SYNB8802 schematic and *in vitro* activity Schematic depicting SYNB8802 oxalate degradation pathway Abbreviations: CO_2_ = carbon dioxide; Frc = formyl‐CoA transferase; OxdC = oxalate decarboxylase; OxlT = oxalate/formate antiporter; ScaaE3 = oxalyl‐CoA synthetase.
*In vitro* activity. Left *y*‐axis: ^13^C_2_‐oxalate in mM, right *y*‐axis: ^13^C‐formate in mM, *x*‐axis: time in minutes. SYNB8802 (blue, circles, solid line: oxalate, dotted line: formate). The control EcN (pink, triangle, solid line: oxalate, dotted line: formate). Three biological replicates were run and plotted separately.
*In vitro* simulation (IVS). Left *y*‐axis: Rate of oxalate degradation in μmol/h/10^9^ cells. *X*‐axis = time in hours. Left *X*‐axis 0–4 h, Right *X*‐axis 6–48 h. Three biological replicates were run and plotted separately. Data points in the orange box represent incubation in simulated gastric fluid. Data points in the light blue box represent incubation in simulated intestinal fluid. Data points in the pink box represent incubation in simulated colonic fluid.

Next, an oxalate‐consuming strain amenable for clinical development was constructed. This necessitated the chromosomal integration and environmental regulation of all engineered components, inclusion of a biocontainment mechanism, and removal of plasmids and antibiotic resistance cassettes used during strain construction (Charbonneau *et al*, 2020). Chromosomal integration reduces gene copy number, which may reduce strain activity. To address this, the ribosome binding sites of the *oxlT*, *scaaE3*, *oxdC*, and *frc* genes were optimized for high expression (Salis, 2011). Chromosomally integrated genes were placed under the regulatory control of the anaerobic‐inducible promoter (P_fnrS_) and the anaerobic‐responsive transcriptional activator, FNR, to enable de novo production of oxalate‐metabolizing genes *in vivo* (Boysen *et al*, 2010; Durand & Storz, 2010; Isabella *et al*, 2018). As a biocontainment measure, the thymidine synthetase gene, *thyA*, was deleted to create an auxotrophic strain unable to grow without exogenous thymidine supplementation (Kurtz *et al*, 2019). Antibiotic markers introduced into the genome during strain construction were removed, and the resulting strain was designated SYNB8802 (Fig 1A). The novel integrated strain showed improved activity over the plasmid‐based prototype (Fig EV1C). SYNB8802 activity was assessed in media containing ^13^C_2_‐oxalate. While no oxalate consumption or formate production was observed for control EcN, SYNB8802 degraded ^13^C_2_‐oxalate in a linear fashion over the course of 60 min and concurrently produced ^13^C‐labeled formate (Fig 1B). Furthermore, to ensure that SYNB8802 was sufficiently optimized, and further copies of the pathway genes did not increase activity, each individual component was overexpressed in the background of SYNB8802, which did not result in improved oxalate degradation (Fig EV1D).

To estimate SYNB8802 activity under conditions representing the GI lumen, an *in vitro* gastrointestinal simulation (IVS) system was developed, comprising a series of incubations in media representing human stomach, small intestine, and colon compartments by simulating luminal pH and oxygen, gastric and pancreatic enzymes, and GI transit times. The rate of SYNB8802 oxalate degradation was estimated in each simulated compartment (Fig 1C). Oxalate consumption was highest in simulated gastric fluid (SGF) (1.35 ± 0.04 and 1.52 ± 0.08 µmol oxalate/h*10^9^ cells at 1 and 2 h post‐inoculation, respectively) and remained at similar levels after 1 h incubation in simulated small intestinal fluid (SIF). Oxalate consumption decreased to 0.88 ± 0.04 µmol oxalate/h*10^9^ cells after 2 h incubation in SIF. SYNB8802 activity further decreased to 0.2 ± 0.14 µmol oxalate/h*10^9^ cells in the completely anaerobic conditions of simulated colonic fluid (SCF), where it remained relatively stable over the 48‐h incubation period. These data suggest that SYNB8802 has the potential to metabolize oxalate throughout all compartments of the human GI tract.

### SYNB8802 is a non‐colonizing strain *in vivo*

**Figure EV2:**
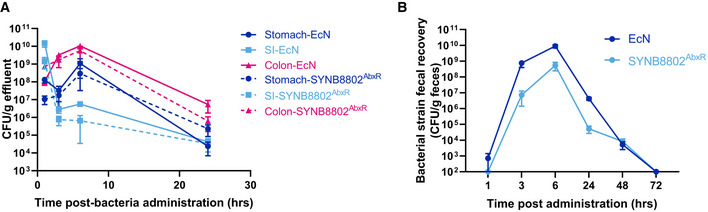
EcN and SYNB8802 intestinal distribution and fecal clearance in mice EcN and SYNB8802^AbxR^ *in vivo* kinetics. Abundance of EcN or SYNB8802^AbxR^ in gut effluents of healthy mice following single oral dose of bacteria. Data represented as mean ± SEM, *n* = 4/group/timepoint. Full lines represent EcN and hatched lines represent SYNB8802^AbxR^.EcN and SYNB8802^AbxR^ fecal clearance. Abundance of EcN or SYNB8802^AbxR^ in fecal pellets of healthy mice following single oral dose of bacteria. Data represented as mean ± SEM, *n* = 5/group. Dark blue curve represents EcN and light blue curve represent SYNB8802^AbxR^.

To determine the *in vivo* viability and gut transit of SYNB8802, an antibiotic‐resistant prototype strain, SYNB8802^AbxR^, was used. SYNB8802^AbxR^ and SYNB8802 have identical genetic modifications, but SYNB8802^AbxR^ has a kanamycin‐resistance cassette to aid in bacterial enumeration in gut effluents and fecal matter. Studies in healthy mice with EcN^StR^ and SYNB8802^AbxR^ demonstrated very similar kinetics and excretion patterns between the strains (Fig EV2). By 1 h, both strains were most abundant in the small intestine and rapidly cleared so that by 24 h, the majority of the cells were found in the colon. When measuring the excretion of EcN and SYNB8802^AbxR^ in the feces, both strains were completely excreted by 72 h, suggesting that the engineering of SYNB8802 did not impact the kinetics and excretion profile as compared to EcN^StR^. Overall, no substantial difference between EcN^StR^ and SYNB8802^AbxR^ was observed in the survival or GI distribution over the duration of the study and this data indicate that, similar to EcN^StR^, SYNB8802^AbxR^ is a non‐colonizing bacterial strain in mice.

### SYNB8802 degrades oxalate *in vivo*

**Figure 2:**
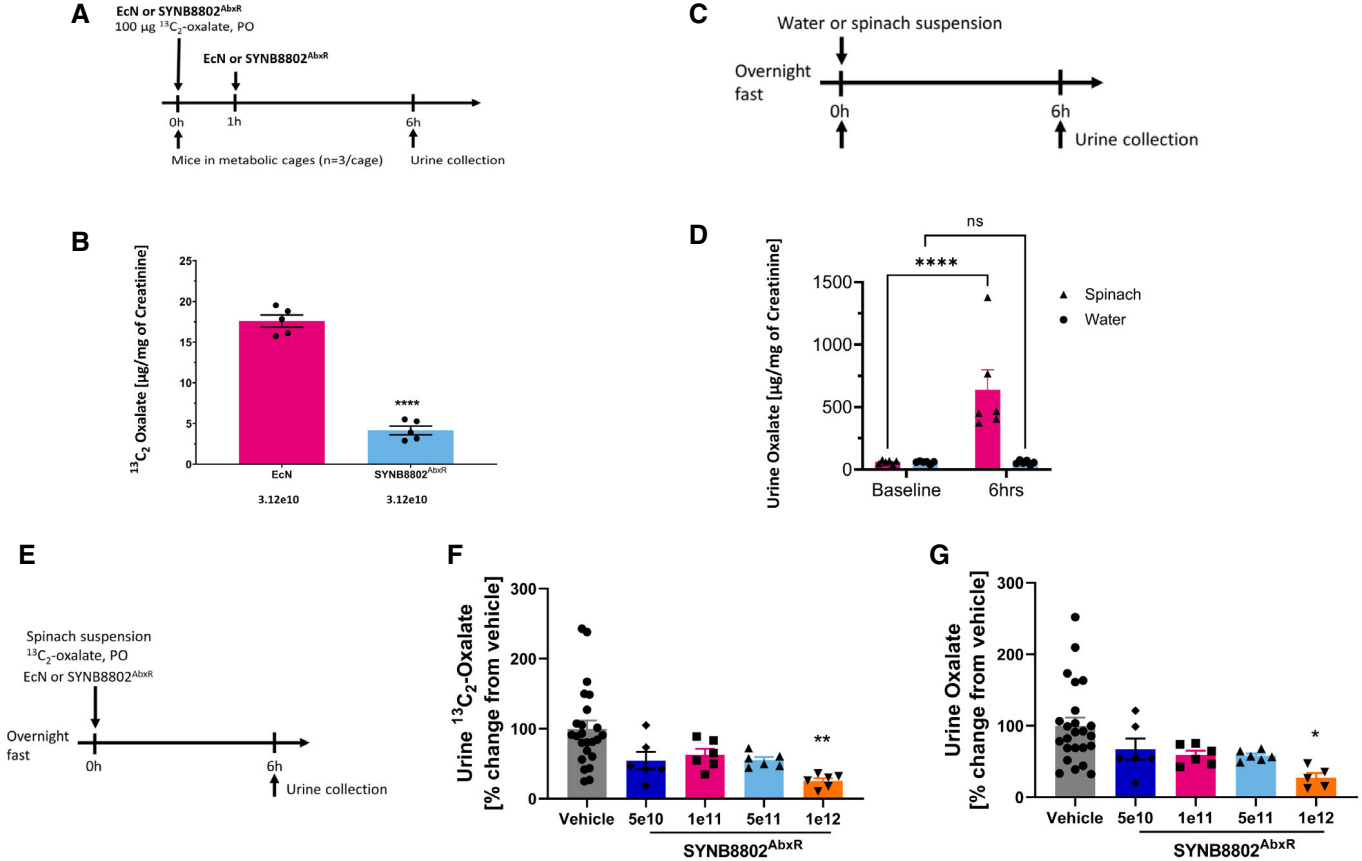
SYNB8802 *in vivo* activity Acute mouse study design.Acute mouse study of UOx lowering with SYNB8802. The *y*‐axis shows the urinary ^13^C_2_‐oxalate normalized to creatine. The *x*‐axis shows the two groups, EcN (control, pink) versus SYNB8802^AbxR^ (light blue; *n* = 15 mice per group). Individual dots represent each metabolic cage (*n* = 3 mice per metabolic cage). Error bars are calculated as SEM (unpaired *t*‐test with Welch's correction, ****P < 0.0001).Non‐human primate model development study design.Non‐human primate model development study. The *y*‐axis shows urinary oxalate (UOx) normalized to creatine. *X*‐axis shows baseline UOx levels after overnight fast, and UOx measured 6 h post‐administration of either a spinach preparation (triangle, pink) or water (circle, blue) (*n* = 6 for each group). Individual dots represent individual animals. Error bars are calculated as SEM (two‐way ANOVA followed by Sidak's multiple comparison analysis, ****P < 0.0001).Study design for urinary recovery of oxalate in non‐human primates.Urinary recovery of oxalate in non‐human primates. The *y*‐axis shows change in UOx from vehicle control. The *x*‐axis shows vehicle (control, grey) and increasing doses of SYNB8802^AbxR^ (*n* = 24 for vehicle, *n* = 6 for treatment groups). Individual dots represent normalized UOx for each individual animal. Error bars are calculated as SEM (One‐way ANOVA followed by Tukey's multiple comparison analysis, *P < 0.05).Urinary recovery of ^13^C_2_ oxalate in non‐human primates. The *y*‐axis shows change in urinary ^13^C_2_‐oxalate from vehicle control. The *x*‐axis shows vehicle (control, grey) and increasing doses of SYNB8802^AbxR^ (*n* = 24 for vehicle, *n* = 6 for treatment groups). Individual dots represent normalized urinary ^13^C_2_‐oxalate for each individual animal. Error bars are calculated as SEM (one‐way ANOVA followed by Tukey's multiple comparison analysis, **P < 0.01).

To assess oxalate consumption *in vivo*, SYNB8802^AbxR^ was co‐administered with ^13^C_2_‐labeled oxalate by oral gavage in mice, and the excretion of labeled oxalate was measured in urine (Fig 2A). Oral administration of SYNB8802^AbxR^ significantly lowered the urinary recovery of ^13^C_2_‐oxalate compared to wild‐type EcN^Str^ (4.14 ± 1.1 vs. 17.59 ± 1.7 μg oxalate/mg creatinine; mean ± SEM, *P* < 0.0001, unpaired *t‐test* with Welch's correction; Fig 2B). This indicates that the engineered strain was active in the GI tract at consuming oxalate and resulted in significantly lowered UOx in mice.

To further characterize *in vivo* activity, an acute model of hyperoxaluria was developed in non‐human primates (NHP). A spinach suspension containing approximately 450 mg oxalate was administered to groups of NHPs to increase UOx levels (Fig 2C). Administration of the spinach suspension resulted in a 10‐fold increase in UOx recovery compared to controls (Fig 2D). Using this model in conjunction with administration of ^13^C_2_‐Oxalate as a non‐dietary source of oxalate (Fig 2E), the ability of SYNB8802^AbxR^ to consume oxalate *in vivo* was evaluated. SYNB8802^AbxR^ lowered unlabeled UOx compared to vehicle by 33, 41, 42, and 73% at the 5 × 10^10^, 1 × 10^11^, 5 × 10^11^, and 1 × 10^12^ CFU dose levels, respectively, with the highest dose achieving statistical significance (one‐way ANOVA followed by Tukey's multiple comparison analysis *P* < 0.05, Fig 2F). A similar relationship was observed for ^13^C_2_‐oxalate excretion, where the 1 × 10^12^ CFU dose significantly lowered ^13^C_2_‐oxalate compared to vehicle (one‐way ANOVA followed by Tukey's multiple comparison analysis *P* < 0.05, Fig 2G). Collectively, these studies indicate that orally administered SYNB8802 significantly lowers UOx levels in mice and NHPs through the consumption of oxalate in the GI tract.

### *In silico* simulation of SYNB8802 activity in the human GI tract

**Figure 3:**
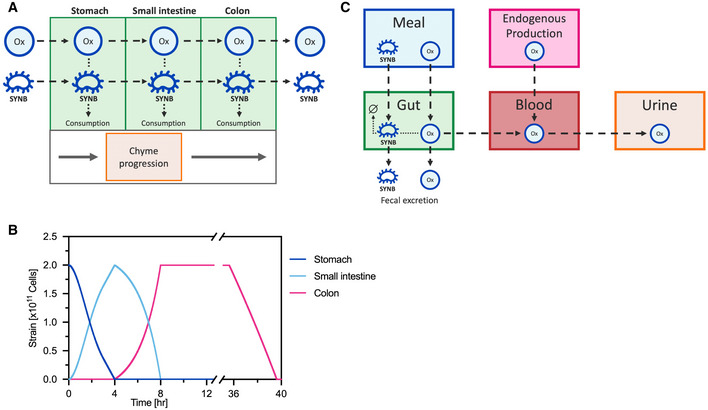
Enteric hyperoxaluria *in silico* simulation (ISS) schematic Oxalate and SYNB8802 transit through the stomach, small intestine, and colon are modeled according to a physiological function of chyme progression. Oxalate absorption and consumption occur sequentially in as chyme transits through the three compartments.SYNB8802 begins in the stomach and empties into the small intestine during the first 4 h post‐meal. From 4 to 8 h post‐meal, SYNB8802 empties from the small intestine into the colon. Approximately 36 h post‐meal, SYNB8802 begins to empty from the colon via fecal excretion.ISS connects *in vitro* strain activity knowledge to host and disease biology. The strain‐side model (green) simulates the consumption of oxalate by SYNB8802 within the gastrointestinal physiology. The host‐side model (overall schematic) simulates the impact of consumption by SYNB8802 on the distribution of oxalate throughout the body.

Mechanistic models combining data from *in vitro* GI simulations and knowledge of human physiology can be used to perform *in silico* simulations (ISS) of synthetic biotic function in the human GI tract (Charbonneau *et al*, 2021). Using this framework, oxalate consumption by SYNB8802 was estimated throughout the GI tract by modeling strain transit sequentially through the stomach, small intestine, and colon (Fig 3A and B). See Materials and Methods for the details of *in silico* model construction. Subsequent effects of SYNB8802 activity on UOx excretion were modeled by considering oxalate absorption into the circulation from the GI tract, endogenous production of oxalate, and urinary excretion (Fig 3C). This multi‐compartment approach also accounted for the anatomical dependence of EH pathophysiology. Since oxalate hyperabsorption in EH occurs primarily in the colon (Dobbins & Binder, 1976; Hylander *et al*, 1978), EH was represented in the ISS model by incorporating an increased rate of oxalate absorption in the colon relative to healthy subjects. Specifically, median predictions assumed four‐fold greater oxalate absorption in EH patients compared to that of healthy subjects, while confidence intervals were based on a range of three‐to‐five times oxalate absorption levels observed in healthy subjects (Chadwick *et al*, 1973; Earnest *et al*, 1974; Modigliani *et al*, 1978; Holmes *et al*, 2001).

**Figure 4:**
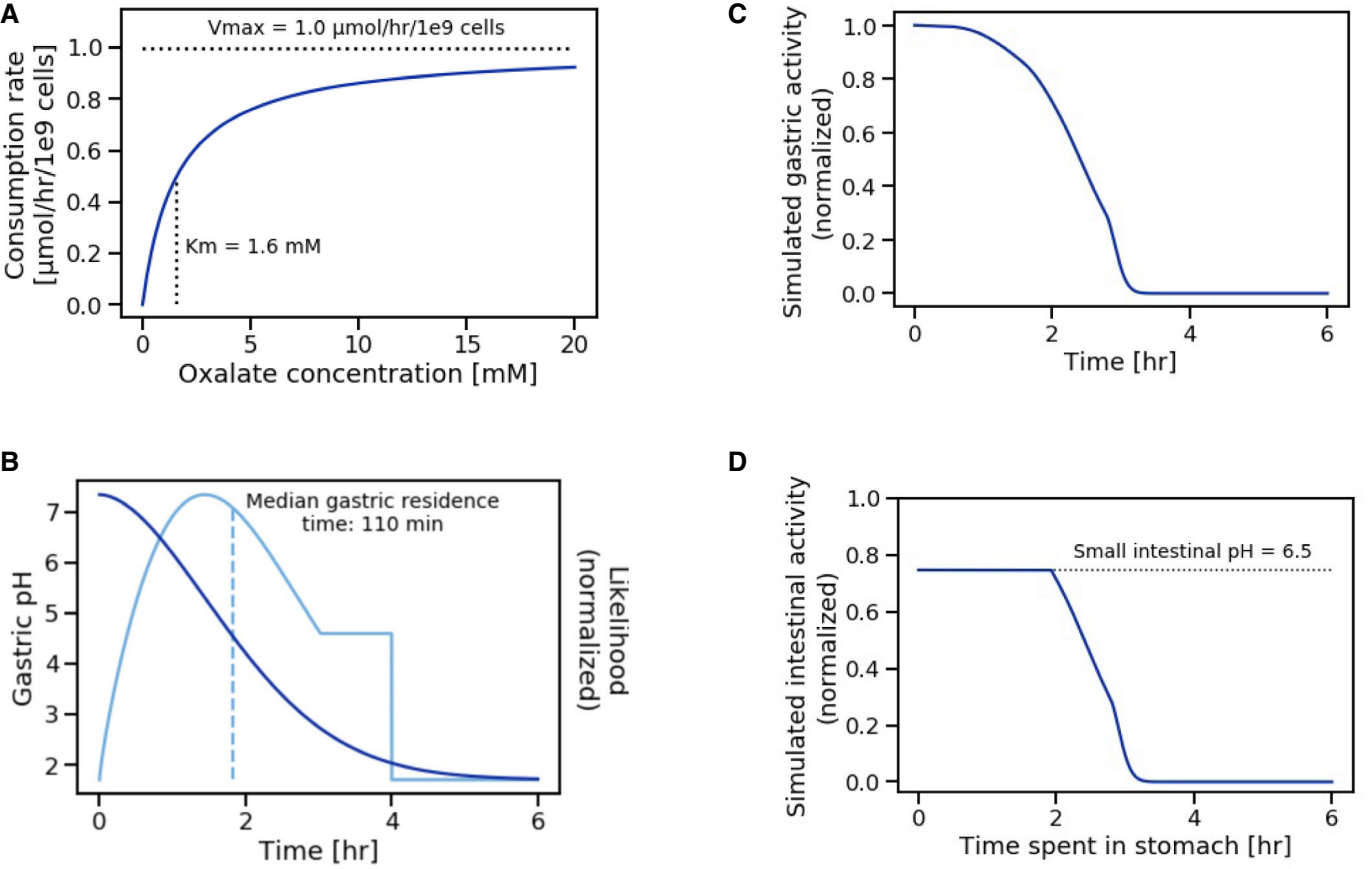
*In silico* simulation (ISS) strain activity and pH inhibition model Michaelis–Menten model of strain activity kinetics. Vmax defines the maximal strain activity velocity (consumption rate of oxalate by SYNB8802). Km defines the oxalate concentration at which half‐maximal strain activity velocity occurs. Vmax and Km were determined through *in vitro* simulation.Simulated gastric pH as a function of time following a solid meal (dark blue). This function is a power exponential decay with a half‐life of 110 min and a shape parameter equal to 1.81. Likelihood of time spent in the stomach based on gastric residence time distribution (light blue). The distribution is truncated to a maximum of 4 h and the median gastric residence time is 110 min.Simulated normalized SYNB8802 activity in the stomach as a function of time.Simulated normalized SYNB8802 activity in the small intestine as a function of time previously spent in the stomach. Function is equivalent to gastric function with an upper limit imposed based on intestinal pH.

**Figure EV3:**
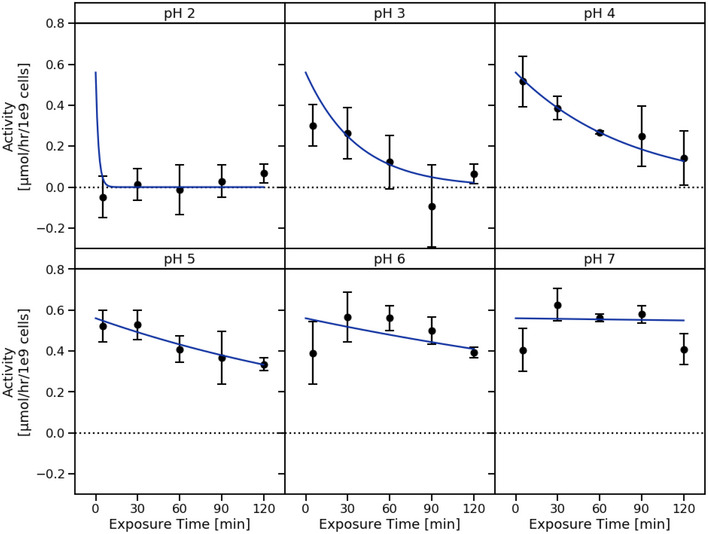
SYNB8802 pH inhibition *in vitro* simulation SYNB8802 activity as a function of exposure time to medium at pH ranging from 2.0 to 7.0. Points and error bars in black represent *in vitro* measurements (*n* = 3 replicate cultures per group; mean ± SD). Blue curves represent exponential decay models fit to *in vitro* measurements for each pH level.

Oxalate consumption by SYNB8802 was modeled according to Michaelis–Menten kinetics by fitting to data from IVS (Fig 4A) while accounting for conditions within the GI tract that may affect strain function. Specifically, oral administration of SYNB8802 involves transient exposure to low pH within the stomach (Gardner *et al*, 2002). Human gastric pH is dynamic, increasing after a meal, then decreasing to ~ 2 in subsequent hours (Fig 4B). In addition, the ISS model of GI transit indicates that a population of SYNB8802 cells in a single dose follows a distribution of gastric residence times (Fig 4B), suggesting that some cells spend more time in the acidic environment of the stomach than others. To understand the effects of environmental pH on oxalate consumption by SYNB8802, an *in vitro* simulation was performed in which SYNB8802 oxalate consumption was determined at a variety of pH levels over time (Fig EV3). Consumption decreased as a function of lower pH and longer exposure times. To account for this observation in the ISS model, an exponential decay function was fit to each pH level (Fig EV3), and these pH effect models were mapped to the dynamic pH of the human stomach *in silico*, such that reduction in strain activity was estimated as a function of time spent in the stomach (Fig 4C). Inhibition of activity due to gastric residence time was then retained for SYNB8802 cells as they transited through the remainder of the GI tract (Fig 4D). Thus, small intestinal and colonic activity of SYNB8802 was informed by how long each individual cell spent in the stomach. Collectively, the ISS model provides a mathematical framework incorporating SYNB8802 activity and information regarding strain and substrate transit through the GI tract to enable physiological estimation of strain performance *in vivo*.

### Validation of simulated UOx excretion

**Figure 5:**
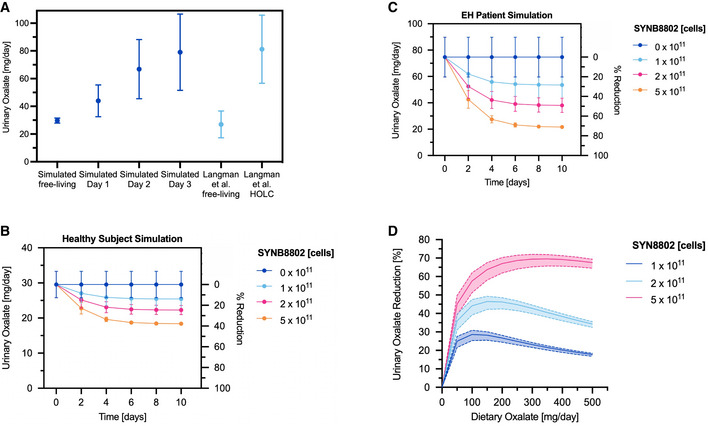
*In silico* simulation (ISS) model validation and simulated urinary oxalate (UOx) lowering subsequent dietary oxalate removal by SYNB8802 Validation of simulated UOx excretion against clinical data. Simulated UOx on a free‐living diet and on 3 days of a high‐oxalate diet (dark blue). Observed UOx on a free‐living diet and on 3 days of a high‐oxalate, low‐calcium (HOLC) diet (light blue); points and error bars represent mean and standard deviation, respectively, across 30 simulated healthy subjects.Simulated UOx and UOx reduction for healthy subjects consuming 200 mg/day dietary oxalate without SYNB8802 and with 1 × 10^11^, 2 × 10^11^, and 5 × 10^11^ SYNB8802 cells TID over 10 days. Points represent simulations under a baseline assumption of dietary oxalate absorption in healthy subjects (Holmes *et al*, 2001) (See Materilas and Methods for a detailed description). Error bars represent a simulated range of dietary oxalate absorption (two simulations: 0.75× baseline and 1.25× baseline).Simulated UOx and UOx reduction for enteric hyperoxaluria patients consuming 200 mg/day dietary oxalate without SYNB8802 and with 1 × 10^11^, 2 × 10^11^, and 5 × 10^11^ SYNB8802 cells TID over 10 days. Points represent simulations under a baseline assumption of increased dietary oxalate absorption in HOX patients (4× healthy absorption). Error bars represent a simulated range of increased dietary oxalate absorption (two simulations: 3× healthy absorption and 5× healthy absorption).Simulated UOx reduction for enteric hyperoxaluria patients after 5 days dosing with 1 × 10^11^, 2 × 10^11^, and 5 × 10^11^ SYNB8802 cells TID as a function of dietary intake of oxalate. Solid curves represent simulations under a baseline assumption of increased dietary oxalate absorption in HOX patients (4× healthy absorption). Shaded regions represent a simulated range of increased dietary oxalate absorption (two simulations: 3× healthy absorption and 5× healthy absorption).

To validate predictions of UOx excretion by the ISS model, simulated data were compared to UOx data from a cohort of 30 healthy subjects (Langman *et al*, 2016). In this study, cumulative 24 h UOx measurements were reported while on a normal diet and after 3 days on a high‐oxalate, low‐calcium (HOLC) diet that increased UOx excretion approximately three‐fold (Fig 5A). Baseline 24 h UOx excretion of healthy subjects was simulated using ISS by assuming dietary oxalate intake of 200 mg/day, resulting in UOx levels of 30 ± 1.8 mg/day (Fig 5A). This result agrees with baseline observations in the population of healthy subjects (27 ± 10 mg/day; Fig 5A) (Langman *et al*, 2016). Next, the effect of an increase in dietary oxalate from 200 to 1,000 mg/day (representing the HOLC diet) was simulated, resulting in an elevation of predicted UOx excretion to 79 ± 28 mg/day on day 3 of the HOLC diet (Fig 5A). Simulated UOx levels on days 2 and 3 post‐elevation of dietary oxalate were consistent with reported UOx levels in healthy subjects on the HOLC diet. Importantly, these simulations relied on no information from the study regarding UOx excretion during the HOLC diet. These data demonstrate that the ISS model accurately describes UOx excretion across a range of dietary oxalate intakes in healthy human subjects.

### ISS model predicts UOx lowering in healthy subjects and EH patients

To estimate the effect of daily SYNB8802 administration on UOx excretion in healthy subjects and EH patients, dosing simulations were performed for three‐times daily doses (TID) of 0, 1 × 10^11^, 2 × 10^11^, or 5 × 10^11^ SYNB8802 cells with meals over 10 days on a diet containing 200 mg/day oxalate. SYNB8802 dose levels were selected based on well‐tolerated doses of related synthetic biotics (Kurtz *et al*, 2019; Puurunen *et al*, 2021). Compared to simulated healthy subjects receiving no therapy, subjects receiving SYNB8802 exhibited lowered UOx excretion after 10 days by 14 ± 3, 25 ± 5, and 38 ± 7% for 1 ×10^11^, 2 × 10^11^, and 5 × 10^11^ cell dose levels, respectively (Fig 5B). In EH patients, simulations show a greater degree of UOx lowering, which was also dose‐dependent: 28 ± 2, 49 ± 3, and 71 ± 4% lowering for 1 × 10^11^, 2 × 10^11^, and 5 × 10^11^ cells, respectively (Fig 5C). The time needed to reach steady state, as well as the relative lowering of the various doses, was similar in healthy subjects and EH patients. An extreme values analysis around select model parameters (Dataset EV4) showed that the magnitude of UOx lowering was robust to alternate physiological assumptions and strain activity kinetics, with the most sensitive parameters being strain activity (maximal strain activity velocity and Michaelis constant) followed by systemic parameters (endogenous production rate and urinary excretion rate constant).

Dietary oxalate intake could substantially influence the effects of SYNB8802, as the strain is restricted to the GI lumen. SYNB8802 UOx lowering was predicted across a range of dietary intakes over 5 days of TID dosing to allow equilibration of serum oxalate, with confidence intervals based on variability in patient physiology as described above (Fig 5D). These simulations suggest that UOx lowering is dependent on dietary oxalate. Notably, maximal UOx lowering was observed at simulated dietary intake levels of 100, 150, and 350 mg/day for 1 × 10^11^, 2 × 10^11^, and 5 × 10^11^ cells, respectively. Importantly, for dietary intakes between 50 and 350 mg/day, all simulated doses were predicted to lower UOx excretion by at least 20%. This range captures the expected daily oxalate intake for individuals on unrestricted diets (Holmes & Kennedy, 2000). Taken together, these results suggest that TID dosing of SYNB8802 with meals could substantially reduce UOx excretion in patients with EH.

## Discussion

The impact of the microbiome in health and diseases has developed into an extensively studied scientific discipline. Members of our microbial community contribute to several metabolic and immune‐mediated diseases, including obesity (Turnbaugh *et al*, 2006), intestinal inflammatory disease (Mazmanian *et al*, 2008), as well as to anti‐cancer immunity (Matson *et al*, 2018). Live bacterial therapeutics are an emerging treatment modality that includes microbiome‐based therapeutics and bacteria engineered to perform specific functions (Landry & Tabor, 2017). The benefit of engineered bacterial strains is the ability to, first, sense and respond to environmental signals within the body (Riglar *et al*, 2017), and second, to program specific functions that be controlled and measured. Bacteria have been engineered for a wide range of therapeutic functions for the treatment of diseases including cancer, immune disorders, and for infection (Sola‐Oladokun *et al*, 2017; Drolia *et al*, 2020). The application of engineered bacteria to metabolic diseases, such as PKU, has involved engineering probiotic bacteria to eliminate toxic metabolites from the GI tract (Isabella *et al*, 2018; Charbonneau *et al*, 2020; Adolfsen *et al*, 2021). In addition to translational models that characterize *in vitro* and *in vivo* strain activity, *in silico* models that can be used to predict strain activity in humans have been developed (Charbonneau *et al*, 2021; Nelson *et al*, 2021). Using these tools, the first live, engineered bacterial therapeutics have been advanced to in Phase 1 and Phase 2 clinical studies (Kurtz *et al*, 2019; Puurunen *et al*, 2021) (NCT04534842).

Reduction in the absorption of oxalate through dietary restriction is a key component of the current approach to EH management. While dietary restriction of oxalate can lower systemic and UOx levels and reduce the occurrence of kidney stones (D'Costa *et al*, 2021), patient adherence is low given the difficulty of restricting high oxalate foods (Attalla *et al*, 2014). Another potential approach to EH management is to directly degrade oxalate within the GI tract, and clinical trials of investigational therapeutics are exploring this approach using microbial (Hoppe *et al*, 2017) (NCT02000219; NCT03938272) and oral enzyme therapies (NCT03456830). SYNB8802, a non‐colonizing engineered strain of EcN, was designed to consume oxalate in the GI tract to prevent its absorption and reduce UOx levels. The engineered approach and non‐colonizing nature of SYNB8802 facilitate repeat dosing in the clinic which enables optimization of therapeutic activity. In addition to the pharmacological benefits, preclusion of colonization should prevent perturbance of the natural microbiome. However, the effect of oxalate removal by SYNB8802 on the microbiota remains to be determined and will be a component of future studies.

Using genes derived from OF and *S. cerevisiae*, a genetic circuit was constructed in EcN to convert oxalate to formate. In an *in vitro* model that simulates transit through the GI tract, SYNB8802 consumed oxalate throughout the length of the human GI tract, differentiating SYNB8802 from enzyme‐based therapies that are predominantly active in the upper GI tract and from OF therapies that are predominantly active in the colon (Stewart *et al*, 2004). Consumption of oxalate in the colon is particularly important for treating EH, since colonic oxalate absorption is a key determinant of disease pathophysiology (Dobbins & Binder, 1977). SYNB8802 also lowered UOx in murine and NHP models, further supporting its potential to degrade dietary‐derived oxalate in a physiologically relevant manner. While oxalate lowering by OF is greater on a per cell basis *in vitro*, the applicability of OF as a potential therapeutic is limited due to its anaerobic life cycle, site of action, and need for colonization (Knight *et al*, 2013). The generation of significant OF biomass for *in vivo* application also appears challenging for drug development. OF generally grows to a OD_600_ = 0.3–0. 4 after multiple days of incubation (Karamad *et al*, 2019). Comparatively, we recently reported well‐tolerated doses of 2E11 in PKU patients, demonstrating the ability to tune activity with dosing (Puurunen *et al*, 2021). The ability to engineer the oxalate metabolizing capability of OF into a bacterial chassis such as EcN enables the development of a potential therapeutic with advantages for drug development.

To further translate SYNB8802 activity *in vitro* to potential clinical activity, an ISS model was developed using human‐specific physiological parameters to simulate oxalate lowering in healthy subjects and EH patients. The model accounts for SYNB8802 and oxalate gut dynamics throughout the gut, with a “gut‐side” model describing the transit of the synthetic biotic and oxalate though the GI tract and a “circulation‐side” model that translates activity within the gut lumen to effects on UOx excretion. The gut‐side model captures the dynamic response of SYNB8802 to its current microenvironment, as well as the cumulative effect of those previously experienced (e.g., transient exposure to acidic gastric pH). The impact of metabolite removal on the disease state, meanwhile, was validated by recapitulation of the relationship between dietary and UOx observed in the clinic. These quantitative mechanistic descriptions were integrated into a single framework for ISS of SYNB8802 dosing. For EH patients consuming oxalate within the range of expected dietary intake, the ISS model predicted a clinically meaningful, dose‐dependent reduction in UOx after dosing with daily administration of SYNB8802. Simulations using a TID dose of 1 × 10^11^ live SYNB8802 cells predicted UOx lowering of >20% for oxalate dietary intakes ranging from 50 to 350 mg/day. In EH patients, a 20% decrease in UOx is associated with a 25% reduction in the annual odds of a future stone event, which in turn could reduce the risk of developing chronic kidney disease (Chuang *et al*, 2020; D'Costa *et al*, 2021). This highlights the significance of this magnitude of oxalate lowering on potential clinical endpoints.

While ISS provides a powerful toolkit for predicting synthetic biotic activity in human subjects, there are some limitations to this approach. For example, parameters of strain activity are informed primarily by *in vitro* simulations of GI physiology, which are a simplified representation of the conditions encountered *in vivo*. Similarly, ISS does not consider the radial distribution of SYNB8802 within the GI lumen, and it is conceivable that oxalate consumption rates vary as a function of cell dispersion into the loosely adherent mucus layer of the colon. Indeed, different rates of replication and resource utilization by *E. coli* were observed between the mucus layer and luminal contents of mice (Li *et al*, 2015). In addition, the present model implementation also does not simulate the effects of dietary calcium on oxalate bioavailability (Liebman & Chai, 1997), and oxalate absorption parameters are derived from experiments in mice. A more accurate model could potentially be achieved by applying human gut‐on‐a‐chip microfluidics technology that incorporates human tissues and their interactions with synthetic biotics (Nelson *et al*, 2021). Lastly, while this ISS approach estimates uncertainty regarding increased oxalate hyperabsorption in EH patients, additional sources of biological variability exist, including anatomical differences present in EH patients who have undergone bariatric surgery (Quercia *et al*, 2014). A platform incorporating these various aspects of strain function and EH biology could further increase confidence in ISS model predictions across patient populations.

This study describes the development of a new synthetic biotic for the treatment of EH through removal of oxalate in the gut. Synthetic biology, IVS, animal models, and ISS were used to determine the performance of SYNB8802 under conditions that approximate the human GI environment. These approaches suggest that SYNB8802 may achieve a clinically meaningful, dose‐dependent lowering of UOx in EH patients. SYNB8802 is under evaluation in a Phase 1 study of healthy subjects and EH patients to determine safety and ability to lower UOx excretion (NCT04629170). The results will give further insight into SYNB8802's potential as a therapeutic and enable further refinement of the ISS approach. Taken together, these findings represent a significant advance in combining preclinical data and *in silico* modeling to drive the rapid development of a synthetic biotic from conception to first‐in‐human clinical study.

## Materials and Methods

### Strain construction

**Figure EV4:**
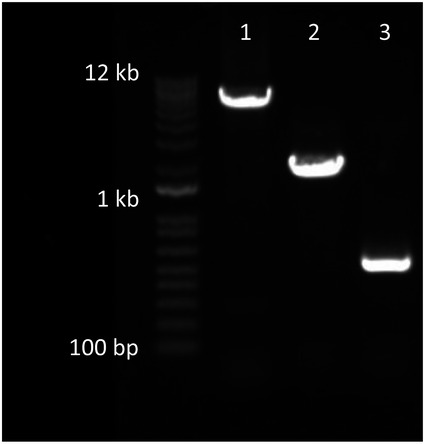
Agarose gel of PCR verification for SYNB8802 Lane 1 shows insertion of *agaI*/*rsmI*:: P_FnrS_ ‐s*caaE3*‐*oxdC*‐*frc*. Lane 2 shows insertion of *exo*/*cea*::P_FnrS_‐*oxlT*. Lane 3 shows knock out *thyA*.

See Table EV1 for a list of strains used in this report. *Escherichia coli* Nissle 1917 (EcN) was purchased from the German Collection of Microorganisms and Cell Cultures (DSMZ Braunschweig, *E. coli* DSM 6601). SYNB8802 was created by insertion of o*xlT* (oxalate:formate transporter [Uniprot ID Q51330; Protein accession: WP_005881678; *O. formigenes* OXCC13]); *frc* (Formyl‐CoA:oxalate CoA‐transferase [EC 2.8.3.16; Uniprot ID C3X9Y2, Protein accession: WP_005880857; *O. formigenes* OXCC13]); *oxdC* (Oxalyl‐CoA decarboxylase [EC 4.1.1.8, Protein accession: WP_005881708; *O. formigenes* OXCC13]); and *scaaE3* (Oxalate:CoA ligase [EC 6.2.1.8; *S. cerevisiae* acyl‐activating enzyme 3; Protein accession: P38137]) into the EcN chromosome. The intergenic loci, *agaI*/*rsmI* and *exo*/*cea* were all identified as suitable integration sites. The aforementioned intergenic regions consist of divergent or convergent promoters separated by a significant length of DNA such that any inserted sequences would not be expected to disrupt the expression of neighboring genes or to be affected by transcription from native adjacent promoters. *OxlT* was integrated under the regulatory control of an anaerobic‐inducible promoter (P_fnrS_) and the anaerobic‐responsive transcriptional activator FNR into the *exo*/*cea* locus (Boysen *et al*, 2010; Durand & Storz, 2010). *ScaaE3*, *oxdC*, and *frc* were integrated under the regulatory control of an anaerobic‐inducible promoter (P_fnrS_) and the anaerobic‐responsive transcriptional activator FNR into the *agaI*/*rsmI* locus. Chromosomal insertions into the EcN genome were performed using the well‐characterized lambda Red recombineering approach (Datsenko & Wanner, 2000). Electroporation was used to introduce DNA for chromosomal modification in EcN (Eporator, Eppendorf, 1.8‐kV pulse, 1‐mm gap length electro‐cuvettes), and transformed cells were selected as colonies on LB agar (Sigma, L2897) containing carbenicillin at 100 µg/ml or kanamycin at 50 µg/ml or chloramphenicol 30 µg/ml where appropriate. For each insertion (i) a pKD3 or pKD4‐based plasmid containing 1,000 bp of 5′ and 3′ EcN genome homology for recombination was built, followed by (ii) insertion of the gene/promoter of interest into the plasmid by isothermal assembly (HiFI DNA Assembly Master Mix, NEB), (iii) amplification of the insertion fragment from the plasmid by PCR (including EcN homology regions and a chloramphenicol or kanamycin resistance cassette) (Platinum SuperFI PCR Master Mix, ThermoFisher Scientific), (iv) recombineering of the insertion fragment by electroporation via pKD46 and subsequent pKD46 removal, and (v) the removal of antibiotic resistance cassettes via pCP20 and subsequent pCP20 removal. All DNA sequences for genomic insertions used in the construction of SYNB8802 are available upon request. Primer sequences are available in Table EV2. For deletion of the *thyA* gene, primers with 40 bp overhang targeting *thyA* were used and pKD3 was used as the template DNA (Datsenko & Warner, 2000). The primers were designed to generate a dsDNA fragment that contained homology adjacent to the *thyA* gene locus in the EcN chromosome and a chloramphenicol resistance gene flanked by flippase recognition target (frt) sites. EcN containing pKD46 was transformed with the *thyA* knockout fragment by electroporation. Colonies were selected on LB agar containing chloramphenicol (30 µg/ml) and thymidine (Sigma, CAS 50‐89‐5; 3 mM). Finally, pcp20 was used to remove the chloramphenicol cassette utilized in the *thyA* knockout, to generate the final, antibiotic‐free strain, SYNB8802. PCR verification (Table EV2, Fig EV4) was run on the final strain SYNB8802 and verified via sanger sequencing. The WGS of the final strain can be accessed here: BioProject: PRJNA780099, Accession number: CP087958.

### Growth and induction of strains

#### *In vitro* assays

All test articles were prepared and induced in shake flasks (Table EV1). Cells were thawed from a frozen (< −65°C) cell bank and grown overnight in 2 ml of LB (Difco™ LB Broth, Miller, Cat. No. 244620) containing appropriate antibiotics and/or supplements in a 14 ml culture tube (Corning Falcon® 14 ml Round Bottom, product number 352059) at 37°C with shaking at 250 rpm. The next day, cell cultures were diluted 1:100 in 50 ml in fresh LB in 250 ml baffled flasks at 37°C with shaking at 250 rpm for 2 h, also with appropriate antibiotics and/or supplements. At this point, the cells were induced by placing flasks in an anaerobic chamber (Coy Laboratory Products, Inc., Grass Lake, MI) supplying an atmosphere of 85% nitrogen, 10% carbon dioxide, and 5% hydrogen. The flask‐based inductions proceeded for 4 h. Following induction, cells were concentrated by centrifugation at 4,000 *g*, resuspended to 500 µl formulation buffer (PBS + 25% Glycerol), and stored at −65°C.

#### Oxalobacter formigenes

*Oxalobacter formigenes* OxB (ATCC part #35274) was grown statically at 37°C in Schaedler Broth (BD Biosciences) supplemented with 100 mM sodium oxalate and 10 mM sodium acetate in an anaerobic chamber (Coy laboratories, Grass Lake, MI) supplying an atmosphere of 85% nitrogen, 10% carbon dioxide, and 5% hydrogen. The medium was autoclaved and placed in the anaerobic chamber at least overnight before use. Cultures started with actively growing cells at OD_600_ 0.02–0.03 and incubated for 48 h at 37°C. Cells were harvested by centrifugation for 8 min at 7,500 *g* at 4°C and concentrated by approximately 500‐fold. The cells were resuspended in formulation buffer (PBS + 25% glycerol) and stored at −80°C.

#### IVS and *in vivo* studies

Cells were thawed from a frozen (< −65°C) cell bank and grown overnight in fermentation media, which was prepared as followed: Yeast extract (40 g/l), K_2_HPO_4_ (5 g/l), KH_2_PO_4_ (3.5 g/l), (NH4)_2_HPO_4_ (3.5 g/l), MgSO_4_*7H_2_O (0.5 g/l), FeCl_3_ (1.6 mg/l), CoCl_2_*6H_2_O (0.2 mg/ml), CuCl_2_ (0.1 mg/l), ZnCl_2_ (0.2 mg/l), NaMoO_4_ (0.2 mg/l), H_3_BO_3_ (0.05 mg/l), Antifoam 204 (125 μl/l), Galactose (30 g/l), Thymidine (20 mM). Cells were grown at 37°C with shaking at 350 rpm. The next day cultures were back diluted to a starting OD of 0.18 and grown in modified fermentation media (Yeast extract [40 g/l], K_2_HPO_4_ [5 g/l], KH_2_PO_4_ [3.5 g/l], (NH4)_2_HPO_4_ [3.5 g/l], MgSO_4_*7H_2_O [0.5 g/l], FeCl_3_ [1.6 mg/l], CoCl_2_*6H_2_O [0.2 mg/ml], CuCl_2_ [0.1 mg/l], ZnCl_2_ [0.2 mg/l], NaMoO_4_ [0.2 mg/l], H_3_BO_3_ [0.05 mg/l], Antifoam 204 [125 μl/l], Galactose [30 g/l], Thymidine [20 mM], Sodium Formate [0.35 g/l], and Sodium Furmarate [6 g/l]) and induced for activity in a fully controlled fermenter system to high cell density followed by washing, concentration, reformulation, and lyophilization. This process simulates the production of drug product intended for human dosing.

#### *In vitro* oxalate degradation assay

Strains were grown in shake flasks and subsequently activated in an anaerobic chamber followed by concentration and freezing at ≤ −65^○^C in glycerol‐based formulation buffer (PBS + 25% Glycerol). In assay media (M9 minimal media, 0.125% d‐Arabinose, 0.125% d‐Galactose, 0.125% d‐Gluconate, and 0.125% d‐Fucose) containing 10 mM ^13^C_2_‐oxalate, activated cells were resuspended to OD_600_ = 5 and incubated statically at 37^○^C for all *in vitro* assays. However, for the experiment comparing SYNB8802 to *O*. *formigenes*, cells were resuspended to an OD_600_ = 0.05. Supernatant samples were removed at indicated timepoints to determine the concentrations of ^13^C_2_‐oxalate and ^13^C‐formate. The concentrations of ^13^C_2_‐oxalate and ^13^C‐formate were determined by liquid chromatography‐tandem mass spectrometry (LC‐MS/MS).

### *In vitro* GI simulations (IVS) assay

#### Simulated gastric fluid incubations

Cells were thawed at room temperature from a frozen (< −65°C) cell bank and diluted in a 0.077 M sodium bicarbonate buffer solution at pH 7.0 in a 96‐well deep‐well plate at 5 × 10^9^ cells/ml. The plate was then transferred into a microaerobic chamber (Coy Laboratory Products, Inc., Grass Lake, MI) calibrated to 7% oxygen and 37°C. Inside the chamber, the bicarbonate and cell suspension was mixed 1:1 with a SGF which is comprised of buffering salts, pepsin enzyme, peptone, mixed sugars (galactose, fucose, arabinose, and gluconate), and 10 mM of Potassium Oxalate Monohydrate (Minekus *et al,* 2014) (for full composition see Dataset EV1). After mixing, the cells in SGF were incubated for 2 h with agitation at 250 rpm for 2 h with cell supernatants collected at 0, 1, and 2 h. During the 2‐h incubation, the chamber was gradually decreased to 4% oxygen. All supernatant samples were stored at −80°C prior to oxalate measurements by LC‐MS/MS.

#### Simulated intestinal fluid (SIF) incubations

Cells were thawed at room temperature from a frozen (< −65°C) cell bank and diluted in a 0.077 M sodium bicarbonate buffer solution at pH 7.0 in a 96‐well deep‐well plate at 6 × 10^10^ cells/ml. The plate was then transferred into a microaerobic chamber (Coy Laboratory Products, Inc., Grass Lake, MI) calibrated to 7% oxygen and 37°C and mixed 1:1 with SGF. After mixing, the cells in SGF were incubated for 2 h with agitation at 250 rpm for 2 h. During the 2 h incubation, the chamber was gradually decreased to 4% oxygen. After 2 h incubation, the solution was then mixed again 1:1 with SIF which is comprised of buffering salts, pancreatin enzyme, bile salts, peptone, mixed sugars (same as previously described), and 10 mM of Potassium Oxalate Monohydrate (Minekus *et al*, 2014) (for full composition see Dataset EV1). With a final cell concentration of 2.5 × 10^9^ cells/ml, the plate was then incubated with agitation at 250 rpm for an additional 2 h, with supernatants collected at 0, 1, and 2 h. All supernatant samples were stored at −80°C prior to oxalate measurements by LC‐MS/MS.

#### Simulated colon fluid incubations

SYNB8802 was thawed at room temperature from a frozen (< −65°C) cell bank, diluted to 1 × 10^10^ cells/ml in simulated colon fluid, and transferred to a 37°C incubator within an anaerobic chamber (Coy Laboratory Products, Inc., Grass Lake, MI) under strict anaerobic conditions. In the same anaerobic chamber, aliquots of 100 µl were removed in triplicate and oxalate consumption measured after the addition of an equal volume of simulated colon fluid with 20 mM potassium oxalate monohydrate and a total of 1% w/v galactose, arabinose, fucose, and gluconate (Minekus *et al,* 2014) (for full composition see Dataset EV1). The final oxalate consumption assay conditions under anaerobic conditions at 37°C were 5 × 10^9^ cells/ml SYNB8802, 10 mM oxalate, and a total 0.05% w/v galactose, arabinose, fucose, gluconate in simulated colon fluid. Supernatant samples were collected immediately after mixing and after 60 min. Oxalate concentrations were determined using LC‐MS/MS and the change over time was used to calculate the rate of consumption. Separate oxalate consumption assays were done as above after zero, 6, 16, 24, 40, and 48 h holds in simulated colonic conditions. Simulated colon fluid was refreshed prior to each activity assay at 6 h and greater by centrifugation, removal of supernatant, and addition of fresh media. Aliquots were removed prior to and after media exchange and OD_600_ was measured to account for cell loss; consumption rates per cell were adjusted accordingly.

### Ethical statement

All procedures performed on animals were in accordance with the humane guidelines for ethical and sensitive care by the Institutional Animal Care and Use Committee (IACUC) of the U.S. National Institutes of Health. Procedures and protocols related to mouse studies were reviewed and approved by Mispro Biotech Services' Institutional Animal Care and Use Committee. Standard operating procedures related to NHP studies have been reviewed and approved by Charles River Laboratories' Institutional Animal Care and Use Committee.

### Bacterial strain *in vivo* viability and excretion kinetics

Animals were group housed and acclimated to the animal housing facility for at least 4 days prior to manipulation for study‐related purposes. Animals were maintained in a controlled temperature and humidity environment (12‐h light/dark cycle) and provided with standard housing, ad libitum water and standard chow diet (LabDiet 5053 PicoLab Rodent Diet), and enrichment prior to start of study. Thirty‐two (32) 13‐ to 23‐week‐old male C57BL/6J mice were group housed and assigned to groups (*n* = 16/group) based on average cage body weight. Mice received a single oral dose of EcN^StR^ or SYNB8802^AbxR^ at 1.22 × 10^10^ CFU and were sacrificed (*n* = 4/group/timepoint) by CO_2_ asphyxiation at their assigned time (1, 3, 6, or 24 h). Feces were collected fresh by free catch, and gut effluents were collected from stomach, small intestine, and colon by flushing with 500 µl of PBS. Feces and gut effluents were placed into pre‐weighed bead‐bug tubes containing 500 µl of PBS, weighed, and then processed for serial dilution and plating on kanamycin‐containing plates to enumerate viable colony‐forming units (CFUs) immediately after collection.

### Acute ^13^C_2_‐induced hyperoxaluria in C57BL/6 mice and urinary recovery

Animals were group housed and acclimated to the animal housing facility for at least 4 days prior to manipulation for study‐related purposes. Animals were maintained in a controlled temperature and humidity environment (12‐h light/dark cycle) and provided with standard housing, ad libitum water and standard chow diet (LabDiet 5053 PicoLab Rodent Diet), and enrichment prior to start of study. Sixty (60) 12‐week‐old male C57BL/6J mice were assigned to groups based on body weight (*n* = 15). In this mouse pharmacology study, labeled oxalate was used as a tracer and the dose was selected based on tolerability and feasibility; the intent was not to directly mimic the dietary oxalate levels consumed by EH patients. Mice received an oral dose of 100 µl ^13^C‐oxalate (100 µg) immediately followed by 200 µl of bacteria at 1.25 × 10^10^ CFU; group one received control EcN^Str^ and group two received SYNB8802^AbxR^. Mice were then immediately placed into metabolic cages (*n* = 3 per cage) for urine collection. One (1) hour post first dose, animals were administered a dose of 300 µl bacteria at 1.87 × 10^10^ CFU; group one received control EcN^Str^ and group two received SYNB8802^AbxR^. The combined cells received per animal from the two doses of bacteria totaled 3.12 × 10^10^ CFU. Mice remained in the metabolic racks for urine collection for a total of 6 h with ad libitum access to food and water. Cumulative urine was collected at the 6‐h time point post first dose and analyzed by LC/MS‐MS. A total of five studies were performed following this protocol; data of a representative study is presented in Fig 2A.

### Acute diet‐induced hyperoxaluria in non‐human primates and urinary recovery

Non‐naïve male cynomolgus monkeys (2–5 years of age) were group housed in wire‐mesh floor cages, except for collection time points during study, and maintained in a controlled temperature and humidity environment (12‐h light/dark cycle) with water ad libitum and food provided in amounts appropriate for the size and age of the animals twice daily. Animals were fasted the night prior to the study for approximately 16–18 h. On the morning of the experiment each monkey was removed from its cage and orally administered vehicle (water) or a spinach suspension (1.3 g/ml in water) for model development (Fig 2B), or vehicle (formulation buffer [13.8%w/v Trehalose, 68 mM Tris, 55 mM HCl, 1× phosphate‐buffered saline (PBS)]) or a spinach suspension (1.5 g/ml) with sodium bicarbonate (1.8 mmol), ^13^C_2‐_oxalate (50 mg), and formulation buffer (13.8%w/v Trehalose, 68 mM Tris, 55 mM HCl, 1× PBS) or bacteria. Animals were then returned to their cages and a clean urine collection pan was placed at the bottom of each cage. Urine was collected at 6 h post‐dosing, and the total urine volume was recorded. Cumulative UOx and creatinine levels were measured by LC/MS‐MS. To determine the dose‐response of SYNB8802 in this model, four identical studies conducted on separate days were performed using the same group of 12 animals, and the data from all studies were combined. Group assignment was alternated across studies and data was expressed as percent change from the respective vehicle for the day of the experiment to minimize the day‐to‐day impact on the experimental results.

### LC‐MS/MS performance

^13^C_2_‐oxalate and ^13^C‐formate were quantitated in bacterial supernatant and biological matrices by LC‐MS/MS using a Thermo Vanquish UHPLC‐Altis TSQ MS system. Standards were prepared at 0.8–1,000 µg/ml in water. Samples were diluted two‐fold with water. Samples and standards are diluted 10‐fold with 10 mM ammonium acetate in water that includes 1 µg/ml ^12^C_2_‐oxalate and ^13^C‐d‐formate as internal standards. Ten microliters were injected onto a Waters Acquity HSS T3 1.8 μm 100 A 2.1 × 100 mm column using 10 mM ammonium acetate (A) and methanol (B) at 0.4 ml/min and 50°C. Analytes were separated after an initial 100% A hold for 0.5 min using a gradient from 0 to 95% B over 1.5 min followed by wash and equilibration steps. Compounds were detected by tandem mass spectroscopy with selected reaction monitoring in electrospray negative ion mode using the following ions: ^13^C_2_‐oxalate 91/62, ^12^C_2_‐oxalate 89/61, ^13^C‐formate 46/46, and ^13^C‐d‐formate 47/47.

### Statistical analysis

Group means, standard errors/deviations, and linear regressions were calculated in Microsoft Excel. To calculate *P*‐values, unpaired student's and Welch's *t‐*tests and one‐way ANOVA were performed using Prism 9.1.0.

### Effect of pH exposure on SYNB8802 activity

Cells were thawed at room temperature from a frozen (< −65°C) cell bank. 100 mg each of arabinose, fucose, galactose, and gluconate were added to 80 ml of 0.077 M sodium bicarbonate. The solution was then split into six tubes. Each tube represented one pH hold condition. The pH of each tube was adjusted to represent one pH level: 2.0, 3.0, 4.0, 5.0, 6.0, or 7.0. Cells were then suspended at 1 × 10^10^ cells/ml in triplicate in a 96 deep‐well plate for each condition. While in the bicarbonate suspension, aliquots of cells were pulled at 5, 30, 60, 90, and 120 min from each pH condition and mixed 1 : 1 with neutral pH SGF containing 10 mM Potassium Oxalate Monohydrate. Cell supernatants were then collected at 0, 30, and 60 min and analyzed by LC‐MS/MS for oxalate concentration.

### *In silico* simulation

The modeling approach integrates SYNB8802 activity informed by *in vitro* studies with the GI and circulation physiology to predict UOx lowering by oral administration of SYNB8802. A multi‐compartment approach was taken wherein volume dynamics were modeled alongside SYNB8802 and oxalate dynamics. In contrast to a typical approach assuming static compartment volumes, the volume of chyme, or partially digested food, within each gut organ was considered as the compartment, rather than the organ itself. Plasma oxalate dynamics were modeled as an initial serum level and an eventual steady state resulting from any change in the amount of oxalate absorbed from the gut. This framework allowed for simulation of either increased gut absorption (e.g., introduction of a high‐oxalate diet) or decreased gut absorption (e.g., introduction of SYNB8802). SYNB8802 and oxalate were simulated to enter the stomach with a meal three times per day and progress through the stomach, small intestine, and colon concurrently with chyme. The processes governing oxalate abundance in the gut were described using material balances implemented as ordinary differential equations (ODEs) (equations ((1), (2), (3), (4), (5), (6), (7), (8), (9))). Each ODE describes the rate of change of a state variable from its initial value to the end of the simulation time (48 h). The initial values of all state variables can be found in Dataset EV2 and the parameter values can be found in Dataset EV3. The initial value of the gastric chyme volume state variable was equal to the total gastric emptying volume, taken as the volume of food eaten and fluid drunk per day for a typical human (Sherwood, 1997) divided by the number of meals per day. The total secretions volume was taken as the volume of plasma secretions into the small intestine per day for a typical human (Sherwood, 1997) divided by the number of meals per day. The total intestinal and colonic emptying volumes were based on the values reported by Sherwood *et al* and those reported by Sandle (1998). The intestinal transit time was taken from a study on chyme transit in the gut (Thomas, 2006). The colonic transit time was taken from a study on methods for measuring colonic transit (Southwell *et al*, 2009).

The gastric chyme volume balance is given by equation (1), where $\frac{dV_{\mathrm{gastric}}}{\mathrm{dt}}$ defines the rate of change of the gastric chyme volume and $r_{\mathrm{gastric}\;\mathrm{emptying}}$ defines the rate of chyme emptying from the stomach into the small intestine. The intestinal chyme volume balance is given by equation (2), where $\frac{dV_{\mathrm{SI}}}{\mathrm{dt}}$ defines the rate of change of the intestinal chyme volume, $r_{\mathrm{secretions}}$ defines the rate of fluid secretion from the plasma into the small intestine, $r_{\mathrm{SI}\;\mathrm{fluid}\;\mathrm{abs}}$ defines the rate of fluid absorption from the small intestine into plasma, and $r_{\mathrm{SI}\;\mathrm{emptying}}$ defines the rate of chyme emptying from the small intestine into the colon. The colonic chyme volume balance is given by equation (3), where $\frac{dV_{\mathrm{colon}}}{\mathrm{dt}}$ defines the rate of change of the colonic chyme volume, $r_{\mathrm{colon}\;\mathrm{fluid}\;\mathrm{abs}}$ defines the rate of fluid absorption from the colon into plasma, and $r_{\mathrm{colon}\;\mathrm{emptying}}$ defines the rate of chyme emptying from the colon into feces. All terms in all chyme balances are defined in units of ml/min.

(1) $$\frac{dV_{\mathrm{gastric}}}{\mathrm{dt}}=-r_{\mathrm{gastric}\;\mathrm{emptying}}$$

(2) $$\frac{dV_{\mathrm{SI}}}{\mathrm{dt}}=r_{\mathrm{gastric}\;\mathrm{emptying}}+r_{\mathrm{secretions}}-r_{\mathrm{SI}\;\mathrm{fluid}\;\mathrm{abs}}-r_{\mathrm{SI}\;\mathrm{emptying}}$$

(3) $$\frac{dV_{\mathrm{colon}}}{\mathrm{dt}}=r_{\mathrm{SI}\;\mathrm{emptying}}-r_{\mathrm{colon}\;\mathrm{fluid}\;\mathrm{abs}}-r_{\mathrm{colon}\;\mathrm{emptying}}$$

The gastric oxalate balance is given by equation (4), where $\frac{d{\mathrm{Ox}}_{\mathrm{gastric}}}{\mathrm{dt}}$ defines the rate of change of the gastric oxalate, $r_{\mathrm{gastric}\;\mathrm{oxalate}\;\mathrm{emptying}}$ defines the rate of oxalate emptying from the stomach into the small intestine, and $r_{\mathrm{gastric}\;\mathrm{oxalate}\;\mathrm{cons}}$ defines the rate of oxalate consumption by SYNB8802 in the stomach. The intestinal oxalate balance is given by equation (5), where $\frac{d{\mathrm{Ox}}_{\mathrm{SI}}}{\mathrm{dt}}$ defines the rate of change of the intestinal oxalate, $r_{\mathrm{SI}\;\mathrm{oxalate}\;\mathrm{cons}}$ defines the rate of oxalate consumption by SYNB8802 in the small intestine, $r_{\mathrm{SI}\;\mathrm{oxalate}\;\mathrm{abs}}$ defines the rate of oxalate absorption from the small intestine into plasma, and $r_{\mathrm{SI}\;\mathrm{oxalate}\;\mathrm{emptying}}$ defines the rate of oxalate emptying from the small intestine into the colon. The colonic oxalate balance is given by equation (6), where $\frac{d{\mathrm{Ox}}_{\mathrm{colon}}}{\mathrm{dt}}$ defines the rate of change of the colonic oxalate, $r_{\mathrm{colon}\;\mathrm{oxalate}\;\mathrm{cons}}$ defines the rate of oxalate consumption by SYNB8802 in the colon, $r_{colon\;oxalate\;abs}$ defines the rate of oxalate absorption from the colon into plasma, and $r_{\mathrm{colon}\;\mathrm{oxalate}\;\mathrm{emptying}}$ defines the rate of oxalate emptying from the colon into feces. All terms in all oxalate balances are defined in units of mmol/min.

(4) $$\frac{d{\mathrm{Ox}}_{\mathrm{gastric}}}{\mathrm{dt}}=-r_{\mathrm{gastric}\;\mathrm{oxalate}\;\mathrm{emptying}}-r_{\mathrm{gastric}\;\mathrm{oxalate}\;\mathrm{cons}}$$

(5) $$\frac{d{\mathrm{Ox}}_{\mathrm{SI}}}{\mathrm{dt}}=r_{\mathrm{gastric}\;\mathrm{oxalate}\;\mathrm{emptying}}-r_{\mathrm{SI}\;\mathrm{oxalate}\;\mathrm{cons}}-r_{\mathrm{SI}\;\mathrm{oxalate}\;\mathrm{abs}}-r_{\mathrm{SI}\;\mathrm{oxalate}\;\mathrm{emptying}}$$

(6) $$\frac{d{\mathrm{Ox}}_{\mathrm{colon}}}{\mathrm{dt}}=r_{\mathrm{SI}\;\mathrm{oxalate}\;\mathrm{emptying}}-r_{\mathrm{colon}\;\mathrm{oxalate}\;\mathrm{cons}}-r_{\mathrm{colon}\;\mathrm{oxalate}\;\mathrm{abs}}-r_{\mathrm{colon}\;\mathrm{oxalate}\;\mathrm{emptying}}$$

The gastric SYNB8802 balance is given by equation (7), where $\frac{d{\mathrm{CFU}}_{\mathrm{gastric}}}{\mathrm{dt}}$ defines the rate of change of the gastric SYNB8802 population and $r_{\mathrm{gastric}\;\mathrm{CFU}\;\mathrm{emptying}}$ defines the rate of SYNB8802 emptying from the stomach into the small intestine. The intestinal SYNB8802 balance is given by equation (8), where $\frac{d{\mathrm{CFU}}_{\mathrm{SI}}}{\mathrm{dt}}$ defines the rate of change of the intestinal SYNB8802 population and $r_{\mathrm{SI}\;\mathrm{CFU}\;\mathrm{emptying}}$ defines the rate of SYNB8802 emptying from the small intestine into the colon. The colonic oxalate balance is given by equation (9), where $\frac{d{\mathrm{CFU}}_{\mathrm{colon}}}{\mathrm{dt}}$ defines the rate of change of the colonic SYNB8802 population and $r_{\mathrm{colon}\;\mathrm{CFU}\;\mathrm{emptying}}$ defines the rate of SYNB8802 emptying from the colon into feces. All terms in all SYNB8802 balances are defined in units of cells/min.

(7) $$\frac{d{\mathrm{CFU}}_{\mathrm{gastric}}}{\mathrm{dt}}=-r_{\mathrm{gastric}\;\mathrm{CFU}\;\mathrm{emptying}}$$

(8) $$\frac{d{\mathrm{CFU}}_{\mathrm{SI}}}{\mathrm{dt}}=r_{\mathrm{gastric}\;\mathrm{CFU}\;\mathrm{emptying}}-r_{\mathrm{SI}\;\mathrm{CFU}\;\mathrm{emptying}}$$

(9) $$\frac{d{\mathrm{CFU}}_{\mathrm{colon}}}{\mathrm{dt}}=r_{\mathrm{SI}\;\mathrm{CFU}\;\mathrm{emptying}}-r_{colon\;CFU\;emptying}.$$

Chyme transit from the stomach to small intestine was modeled according to a power exponential decay function for stomach volume based on the work of Elashoff *et al* (1982; Table 1). The rate of gastric emptying was given by $‐V_{\mathrm{total}\;\mathrm{gastric}\;\mathrm{emptying}}\;*\;\frac{\beta \;*\;\ln2*t^{\beta ‐1}}{{\tau_{1/2}}^{\beta }}\;*\;2^{‐{\left(\frac{t}{\tau_{1/2}}\right)}^{\beta }}$, equal to the product of the total gastric emptying volume $V_{\mathrm{total}\;\mathrm{gastric}\;\mathrm{emptying}}$ and the time derivative of the power exponential defined by the half gastric emptying time $\tau_{1/2}$ and the shape parameter β. The gastric emptying function was then modified to terminate in a finite amount of time given by $\tau_{\mathrm{gastric}}$; to achieve this, gastric emptying was switched from power exponential to linear at time $\tau_{\mathrm{linear}}$, then defined as zero from $\tau_{\mathrm{gastric}}$ onward (equation (10)). The rate of fluid secretion from the plasma into the small intestine was modeled as proportional to the gastric chyme emptying rate and the ratio of the total secretions volume $V_{\mathrm{total}\;\mathrm{secretions}}$ to the total gastric emptying volume (equation (11)). The first portion of chyme to exit the stomach was assumed to also be the first to reach the ileocecal valve and empty from the small intestine into the colon; therefore, intestinal emptying begins at the intestinal transit time $\tau_{\mathrm{SI}}$. Likewise, the last portion of chyme to exit the stomach was last to empty from the small intestine and marked the end of the intestinal emptying window at time $\tau_{\mathrm{SI}}+\tau_{\mathrm{gastric}}$. The timeframe of colonic emptying was similarly defined as $\tau_{\mathrm{SI}}+\tau_{\mathrm{colon}}\leq t<\tau_{\mathrm{SI}}+\tau_{\mathrm{colon}}+\tau_{\mathrm{gastric}}$, where $\tau_{\mathrm{colon}}$ defines the colonic transit time. Intestinal and colonic chyme emptying were assumed to be constant during the relevant timeframes and zero otherwise, with the magnitude of the emptying rate equal to the total intestinal or colonic emptying volume $V_{\mathrm{total}\;\mathrm{SI}\;\mathrm{emptying}}$, $V_{\mathrm{total}\;\mathrm{colon}\;\mathrm{emptying}}$ divided by the length of the timeframe (equations (12 and 13)).

(10) $$r_{\mathrm{gastric}\;\mathrm{emptying}}=\left\{\begin{array}{cc} -V_{\mathrm{total}\;\mathrm{gastric}\;\mathrm{emptying}}\;*\;\frac{\beta *\ln_{2}*t^{\beta -1}}{{\tau_{1/2}}^{\beta }}\;*\;2^{-{\left(\frac{t}{\tau_{1/2}}\right)}^{\beta }} & \mathrm{if}\;t<\tau_{\mathrm{linear}}\\ \frac{V_{\mathrm{gastric}}\left(t=\tau_{\mathrm{linear}}\right)}{\tau_{\mathrm{gastric}}-\tau_{\mathrm{linear}}} & \mathrm{if}\;\tau_{\mathrm{linear}}\leq t<\tau_{\mathrm{gastric}}\\ 0 & \mathrm{if}\;t\geq \tau_{\mathrm{gastric}} \end{array}\right.$$

(11) $$r_{\mathrm{secretions}}=r_{\mathrm{gastric}\;\mathrm{emptying}}\;*\;\frac{V_{\mathrm{total}\;\mathrm{secretions}}}{V_{\mathrm{total}\;\mathrm{gastric}\;\mathrm{emptying}}}$$

(12) $$r_{\mathrm{SI}\;\mathrm{emptying}}=\left\{\begin{array}{cc} \frac{V_{\mathrm{total}\;\mathrm{SI}\;\mathrm{emptying}}}{\tau_{\mathrm{gastric}}} & \mathrm{if}\;\tau_{\mathrm{SI}}\leq t<\tau_{\mathrm{SI}}+\tau_{\mathrm{gastric}}\\ 0 & \mathrm{otherwise} \end{array}\right.$$

(13) $$r_{\mathrm{colon}\;\mathrm{emptying}}=\left\{\begin{array}{cc} \frac{V_{\mathrm{total}\;\mathrm{colon}\;\mathrm{emptying}}}{\tau_{\mathrm{gastric}}} & \mathrm{if}\;\tau_{\mathrm{SI}}+\tau_{\mathrm{colon}}\leq t<\tau_{\mathrm{SI}}+\tau_{\mathrm{colon}}+\tau_{\mathrm{gastric}}\\ 0 & \mathrm{otherwise} \end{array}\right..$$

Oxalate emptying from the stomach, small intestine, and colon were defined as the product of the chyme emptying rate in each compartment and the oxalate concentration, equal to the oxalate abundance in mmol divided by the chyme volume in ml (equations ((14), (15), (16))). SYNB8802 emptying was also defined as proportional to chyme transit and SYNB8802 concentration, equal to the SYNB8802 abundance in cells/min divided by the chyme volume in ml (equations ((17), (18), (19))). The rates of fluid absorption from the small intestine and colon into plasma were modeled as first‐order, as the product of the chyme volume and a first‐order kinetic rate constant for fluid absorption $k_{\mathrm{SI}/\mathrm{colon}\;\mathrm{fluid}\;\mathrm{abs}}$ (equations (20 and 21)). The rates of oxalate absorption from the small intestine and colon into plasma were also modeled as first‐order, as the product of the oxalate abundance and a first‐order kinetic rate constant for oxalate absorption $k_{\mathrm{SI}/\mathrm{colon}\;\mathrm{oxalate}\;\mathrm{abs}}$ (equations (22 and 23)).

(14) $$r_{\mathrm{gastric}\;\mathrm{oxalate}\;\mathrm{emptying}}=r_{\mathrm{gastric}\;\mathrm{emptying}}\;*\;\frac{{\mathrm{Ox}}_{\mathrm{gastric}}}{V_{\mathrm{gastric}}}$$

(15) $$r_{\mathrm{SI}\;\mathrm{oxalate}\;\mathrm{emptying}}=r_{\mathrm{SI}\;\mathrm{emptying}}\;*\;\frac{{\mathrm{Ox}}_{\mathrm{SI}}}{V_{\mathrm{SI}}}$$

(16) $$r_{\mathrm{colon}\;\mathrm{oxalate}\;\mathrm{emptying}}=r_{\mathrm{colon}\;\mathrm{emptying}}\;*\;\frac{{\mathrm{Ox}}_{\mathrm{colon}}}{V_{\mathrm{colon}}}$$

(17) $$r_{\mathrm{gastric}\;\mathrm{CFU}\;\mathrm{emptying}}=r_{\mathrm{gastric}\;\mathrm{emptying}}\;*\;\frac{{\mathrm{CFU}}_{\mathrm{gastric}}}{V_{\mathrm{gastric}}}$$

(18) $$r_{\mathrm{SI}\;\mathrm{CFU}\;\mathrm{emptying}}=r_{\mathrm{SI}\;\mathrm{emptying}}\;*\;\frac{{\mathrm{CFU}}_{\mathrm{SI}}}{V_{\mathrm{SI}}}$$

(19) $$r_{\mathrm{colon}\;\mathrm{CFU}\;\mathrm{emptying}}=r_{\mathrm{colon}\;\mathrm{emptying}}\;*\;\frac{{\mathrm{CFU}}_{\mathrm{colon}}}{V_{\mathrm{colon}}}$$

(20) $$r_{\mathrm{SI}\;\mathrm{fluid}\;\mathrm{abs}}=k_{\mathrm{SI}\;\mathrm{fluid}\;\mathrm{abs}}\;*\;V_{\mathrm{SI}}$$

(21) $$r_{\mathrm{colon}\;\mathrm{fluid}\;\mathrm{abs}}=k_{\mathrm{colon}\;\mathrm{fluid}\;\mathrm{abs}}\;*\;V_{\mathrm{colon}}$$

(22) $$r_{\mathrm{SI}\;\mathrm{oxalate}\;\mathrm{abs}}=k_{\mathrm{SI}\;\mathrm{oxalate}\;\mathrm{abs}}\;*\;{\mathrm{Ox}}_{\mathrm{SI}}$$

(23) $$r_{\mathrm{colon}\;\mathrm{oxalate}\;\mathrm{abs}}=k_{\mathrm{colon}\;\mathrm{oxalate}\;\mathrm{abs}}\;*\;{\mathrm{Ox}}_{\mathrm{colon}}.$$

Oxalate consumption by SYNB8802 in each gut compartment was simulated according to the Michaelis–Menten model of enzyme kinetics (Fig 4A). This model defines the rate of consumption as a maximal strain activity velocity $V_{\max}$ times the substrate concentration divided by the sum of a Michaelis constant $K_{M}$ and the substrate concentration; this quantity was then multiplied by the SYNB8802 abundance in each compartment. SYNB8802 activity in the stomach was further modified by a gastric pH inhibition function $K_{\mathrm{gastric}\;\mathrm{pH}\;\mathrm{inhibition}}$ and a gastric oxygen inhibition function $K_{\mathrm{gastric}\;O_{2}\;\mathrm{inhibition}}$ (equation (24)). SYNB8802 activity in the small intestine was modified by an intestinal pH inhibition function $K_{\mathrm{SI}\;\mathrm{pH}\;\mathrm{inhibition}}$ and an intestinal oxygen inhibition function $K_{\mathrm{SI}\;O_{2}\;\mathrm{inhibition}}$ (equation (25)). SYNB8802 activity in the colon was modified by a colonic pH inhibition function $K_{\mathrm{colon}\;\mathrm{pH}\;\mathrm{inhibition}}$, a colonic oxygen inhibition function $K_{\mathrm{colon}\;O_{2}\;\mathrm{inhibition}}$, and an extended colonic activity term $K_{\mathrm{extended}\;\mathrm{colonic}\;\mathrm{activity}}$ (equation (26)). A physiological function of gastric pH decline following a meal was modeled as a power exponential decay function (Fig 4B, dark blue). A half‐time parameter described the time for half of the total pH decline to occur, and a shape parameter described the degree of variance from a simple exponential model. SYNB8802 cells were modeled to follow a gastric residence time distribution truncated to a maximum of 4 h, with a median gastric residence time of 110 min (Fig 4B, light blue). The pH inhibition functions were informed by the SGF experiment (Fig 4C). An exponential decay function was fit to each pH trial and decay constants were interpolated to simulate non‐integer pH values. The relationship between pH and activity decay was then applied to the function of gastric pH dynamics to yield the gastric pH inhibition function (Fig 4D). The model of gastric emptying dynamics was then used to construct a distribution of gastric residence times which, in combination with the gastric pH inhibition function, was used to simulate lowering of intestinal and colonic SYNB8802 activity due to lasting acid damage, yielding the intestinal and colonic pH inhibition functions (Fig 4E). Cells that spent longer in the stomach were considered less active while in the small intestine and colon. A cap of 75% of maximal activity was imposed on the simulated activity of all cells while in the small intestine and colon, regardless of the time spent in the stomach. This was due to the intestinal/colonic pH of 6.5 and was informed by a function describing instantaneous rather than lasting pH effects, fit to in‐house *in vitro* simulations. Normalized SYNB8802 activities of 4 ± 0.2, 4 ± 0.3, 24 ± 0.7, 28 ± 0.6, 30 ± 0.2, 47 ± 2, 52 ± 2, 71 ± 11, and 100 ± 3% were observed at a pH of 3.0, 3.5, 4.0, 4.5, 5.0, 5.5, 6.0, 6.5, and 7.0, respectively. The oxygen inhibition functions in all gut compartments were modeled according to linear decline from the maximal strain activity observed at 21% oxygen, fit to in‐house *in vitro* simulations. Normalized SYNB8802 activities of 74 ± 5, 79 ± 29, and 100 ± 1% were observed at 0%, 7%, and 21% oxygen, respectively. The extended colonic activity term was informed by the SCF *in vitro* simulation via direct interpolation of consumption rate over time (Fig 1C).

(24) $$r_{\mathrm{gastric}\;\mathrm{oxalate}\;\mathrm{cons}}=\frac{V_{\max}*\left[{\mathrm{Ox}}_{\mathrm{gastric}}\right]}{K_{M}+\left[{\mathrm{Ox}}_{\mathrm{gastric}}\right]}\;*\;{\mathrm{CFU}}_{\mathrm{gastric}}\;*\;K_{\mathrm{gastric}\;\mathrm{pH}\;\mathrm{inhibition}}\;*\;K_{\mathrm{gastric}\;O_{2}\;\mathrm{inhibition}}$$

(25) $$r_{\mathrm{SI}\;\mathrm{oxalate}\;\mathrm{cons}}=\frac{V_{\max}*\left[{\mathrm{Ox}}_{\mathrm{SI}}\right]}{K_{M}+\left[{\mathrm{Ox}}_{\mathrm{SI}}\right]}\;*\;{\mathrm{CFU}}_{\mathrm{SI}}\;*\;K_{\mathrm{SI}\;\mathrm{pH}\;\mathrm{inhibition}}\;*\;K_{\mathrm{SI}\;O_{2}\;\mathrm{inhibition}}$$

(26) $$r_{\mathrm{colon}\;\mathrm{oxalate}\;\mathrm{cons}}=\frac{V_{\max}*\left[{\mathrm{Ox}}_{\mathrm{colon}}\right]}{K_{M}+\left[{\mathrm{Ox}}_{\mathrm{colon}}\right]}\;*\;{\mathrm{CFU}}_{\mathrm{colon}}\;*\;K_{\mathrm{colon}\;\mathrm{pH}\;\mathrm{inhibition}}*\;K_{\mathrm{colon}\;O_{2}\;\mathrm{inhibition}}\;*\;K_{\mathrm{extended}\;\mathrm{colonic}\;\mathrm{activity}}.$$

The total gut absorption into plasma was equal to the sum of intestinal and colonic absorption; gastric absorption was not modeled (equation (27)). The first‐order oxalate absorption rate constants in equations (22 and 23) were calibrated such that the total absorption in absence of SYNB8802 $M_{\mathrm{oxalate}\;\mathrm{abs}}\left(\mathrm{SYNB}8802\;\mathrm{absent}\right)$ was equal to the dietary intake $M_{\mathrm{dietary}\;\mathrm{oxalate}}$ times a dietary absorption fraction $f_{\mathrm{abs}}$ (equation (28)), and that the intestinal portion thereof $M_{\mathrm{SI}\;\mathrm{oxalate}\;\mathrm{abs}}\left(\mathrm{SYNB}8802\;\mathrm{absent}\right)$ was equal to the total times an intestinal fraction $f_{\mathrm{SI}\;\mathrm{abs}}$ describing the site of absorption (equation (29)). The dietary absorption fraction for healthy subjects was based on the work of Holmes *et al* (2001) who observed the relationship between dietary oxalate intake and urinary excretion (Fig 5). Twelve healthy individuals were placed on an oxalate‐free diet for 5 days to establish the urinary excretion under endogenous production alone, then switched to a higher‐oxalate diet ranging from 10 to 250 mg/day. The fraction of dietary oxalate absorbed was calculated as the difference between urinary excretion on the oxalate‐containing and oxalate‐free diets divided by the oxalate content of the diet. The ISS approach presented here fit an exponential function to the observed data to determine dietary absorption fraction for healthy subjects as a function of dietary intake. The dietary absorption fraction for EH patients was assumed to be from three to five times greater than that in healthy subjects (equation (30)) (Chadwick *et al*, 1973; Earnest *et al*, 1974; Modigliani *et al*, 1978; Holmes *et al*, 2001). The site of absorption for healthy subjects was informed by a study of oxalate transport across sections of the mouse gut, which yielded permeability constants for the duodenum, jejunum, ileum, proximal colon, and distal colon. (Davies & Morris, 1993)The ratio of the intestinal to colonic permeability constants was assumed to be equal between mice and humans. The site of absorption for EH patients was assumed such that all additional oxalate absorption occurred in the colon; that is, such that intestinal absorption was equal to that in healthy subjects (equation (31)).

(27) $$M_{\mathrm{oxalate}\;\mathrm{abs}}=M_{\mathrm{SI}\;\mathrm{oxalate}\;\mathrm{abs}}+M_{\mathrm{colon}\;\mathrm{oxalate}\;\mathrm{abs}}$$

(28) $$M_{\mathrm{oxalate}\;\mathrm{abs}}\left(\mathrm{SYNB}8802\;\mathrm{absent}\right)=M_{\mathrm{dietary}\;\mathrm{oxalate}}\;*\;f_{\mathrm{abs}}$$

(29) $$M_{\mathrm{SI}\;\mathrm{oxalate}\;\mathrm{abs}}\left(\mathrm{SYNB}8802\;\mathrm{absent}\right)=M_{\mathrm{oxalate}\;\mathrm{abs}}\left(\mathrm{SYNB}8802\;\mathrm{absent}\right)\;*\;f_{\mathrm{SI}\;\mathrm{abs}}$$

(30) $$3\;*\;f_{\mathrm{abs}\;\mathrm{healthy}}\leq f_{\mathrm{abs}\;\mathrm{EH}}\leq 5\;*\;f_{\mathrm{abs}\;\mathrm{healthy}}$$

(31) $$M_{\mathrm{SI}\;\mathrm{oxalate}\;\mathrm{abs}\;\mathrm{EH}}\left(\mathrm{SYNB}8802\;\mathrm{absent}\right)=M_{\mathrm{SI}\;\mathrm{oxalate}\;\mathrm{abs}\;\mathrm{healthy}}\left(\mathrm{SYNB}8802\;\mathrm{absent}\right).$$

The dynamics of oxalate abundance in the plasma were described using a material balance implemented as an ODE, where $\frac{d{\mathrm{Ox}}_{\mathrm{plasma}}}{\mathrm{dt}}$ defines the rate of change of the plasma oxalate abundance, $r_{\mathrm{plasma}\;\mathrm{influx}}$ defines the rate of oxalate influx into plasma and $r_{\mathrm{urinary}}$ defines the rate of urinary excretion of oxalate (equation (32)). Oxalate influx into plasma was defined as the total gut absorption per meal, as calculated in equation (27), times the number of meals per day $N_{\mathrm{daily}\;\mathrm{meals}}$ plus the rate of endogenous production of oxalate $r_{\mathrm{endogenous}}$ (equation (33)). The rate of endogenous production of oxalate was calculated based on the work of Chadwick *et al* (1973) who observed the urinary excretion of EH patients while on several days of an oxalate‐free diet (Fig 4). Urinary excretion was modeled using first‐order kinetics, as the product of the plasma oxalate abundance and a first‐order kinetic rate constant for urinary excretion (equation (34)). The urinary excretion rate constant was based on the work of Holmes *et al* (2001) who observed the UOx excretion dynamics for healthy subjects transitioning from self‐selected diets to an oxalate‐free diet (Fig 1). The plasma oxalate ODE simplified into an exponential decay form describing how the plasma level ${\mathrm{Ox}}_{\mathrm{plasma}}\left(t\right)$ changed from an initial steady state ${\mathrm{Ox}}_{\mathrm{plasma}\;\mathrm{init}}$ to a new steady state ${\mathrm{Ox}}_{\mathrm{plasma}\;\mathrm{SS}}$ (equation (35)). The new steady state was defined as the plasma influx divided by the urinary excretion rate constant (equation (36)). The initial steady state was similarly defined as a previous plasma influx $r_{\mathrm{plasma}\;\mathrm{influx}\;\mathrm{init}}$ (e.g., before dosing with SYNB8802, under a different dietary intake, or both) divided by the urinary excretion rate constant (equation (37)). By combining equations (34 and 35), it can be shown that urinary excretion followed the same dynamics as plasma level, changing from an initial steady state $r_{\mathrm{plasma}\;\mathrm{influx}\;\mathrm{init}}$ to a new steady state $r_{\mathrm{plasma}\;\mathrm{influx}}$ (equation (38)).

(32) $$\frac{d{\mathrm{Ox}}_{\mathrm{plasma}}}{\mathrm{dt}}=r_{\mathrm{plasma}\;\mathrm{influx}}-r_{\mathrm{urinary}}$$

(33) $$r_{\mathrm{plasma}\;\mathrm{influx}}=M_{\mathrm{oxalate}\;\mathrm{abs}}\;*\;N_{\mathrm{daily}\;\mathrm{meals}}+r_{\mathrm{endogenous}}$$

(34) $$r_{\mathrm{urinary}}=k_{\mathrm{urinary}}\;*\;{\mathrm{Ox}}_{\mathrm{plasma}}$$

(35) $${\mathrm{Ox}}_{\mathrm{plasma}}\left(t\right)={\mathrm{Ox}}_{\mathrm{plasma}\;\mathrm{SS}}+\left({\mathrm{Ox}}_{\mathrm{plasma}\;\mathrm{init}}-{\mathrm{Ox}}_{\mathrm{plasma}\;\mathrm{SS}}\right)\;*\;e^{-k_{\mathrm{urinary}}*t}$$

(36) $${\mathrm{Ox}}_{\mathrm{plasma}\;\mathrm{SS}}=\frac{r_{\mathrm{plasma}\;\mathrm{influx}}}{k_{\mathrm{urinary}}}$$

(37) $${\mathrm{Ox}}_{\mathrm{plasma}\;\mathrm{init}}=\frac{r_{\mathrm{plasma}\;\mathrm{influx}\;\mathrm{init}}}{k_{\mathrm{urinary}}}$$

(38) $$r_{\mathrm{urinary}}\left(t\right)=r_{\mathrm{plasma}\;\mathrm{influx}}+\left(r_{\mathrm{plasma}\;\mathrm{influx}\;\mathrm{init}}-r_{\mathrm{plasma}\;\mathrm{influx}}\right)\;*\;e^{-k_{\mathrm{urinary}}*t}$$

Finally, the results of an extreme values analysis on select parameters of the modeling framework can be found in Dataset EV4. The simulated UOx reduction for enteric hyperoxaluria patients consuming 200 mg/day dietary oxalate after 10 days dosing with 5 × 10^11^ SYNB8802 cells TID, 71.1%, was used as the baseline efficacy. A range of values was taken from the relevant literature for each of the following model parameters: gastric emptying curve shape parameter, half gastric emptying time, intestinal chyme volume fraction, colonic chyme volume fraction, intestinal transit time, colonic transit time, fraction dietary oxalate absorbed in patients, fraction of oxalate absorption occurring in the small intestine, endogenous production rate, and urinary excretion rate constant; and from in‐house *in vitro* simulations for the SYNB8802 maximal strain activity velocity and Michaelis constant. The intestinal chyme volume fraction was defined as the total intestinal emptying volume divided by the sum of the total gastric emptying volume and total secretions volume, while the colonic chyme volume fraction was defined as the total colonic emptying volume divided by the total intestinal emptying volume. The UOx reduction for enteric hyperoxaluria patients consuming 200 mg/day dietary oxalate after 10 days dosing with 5 × 10^11^ SYNB8802 cells TID was simulated under the minimal and maximal values of each of these parameters.

### Software

*In silico* simulations were all implemented in Python 3.7.6, using Jupyter version 6.0.3 (jupyter.org). Ordinary differential equations were solved using SciPy version 1.4.1 (scipy.org). Statistical analysis was performed using Prism 9.1.0 (GraphPad, San Diego, CA). The code for all *in silico* simulations is available for download on Zenodo (Horvath, 2022).

#### Strains described in the study

*Escherichia coli* Nissle strain 1917 was obtained from DSMZ and used for the construction of engineered strains leading to the generation of SYNB8802. All strains described in this manuscript were derived from the same parental background. Engineered strains described in this manuscript can be made available subject to an MTA, which can be requested by contacting the corresponding authors.

##### Life sciences reporting summary

Further information on experimental design is available in the EMBO press author checklist for Life Sciences Articles linked to this manuscript.

## Author contributions

**David Lubkowicz:** Conceptualization; Data curation; Formal analysis; Visualization; Writing—original draft; Writing—review and editing. **Nicholas Horvath:** Conceptualization; Data curation; Software; Formal analysis; Writing—original draft; Writing—review and editing. **Michael J James:** Data curation; Formal analysis; Methodology. **Pasquale Cantarella:** Data curation; Formal analysis; Writing—original draft. **Lauren Renaud:** Data curation; Formal analysis. **Christopher G Bergeron:** Resources; Data curation. **Ron B Shmueli:** Resources; Data curation. **Cami Anderson:** Conceptualization. **Jian‐Rong Gao:** Data curation; Writing—original draft; Writing—review and editing. **Caroline B Kurtz:** Writing—review and editing. **Mylene Perreault:** Visualization; Writing—original draft; Writing—review and editing. **Mark R Charbonneau:** Writing—original draft; Writing—review and editing. **Vincent M Isabella:** Writing—original draft; Project administration; Writing—review and editing. **David L Hava:** Writing—original draft; Project administration; Writing—review and editing.

In addition to the CRediT author contributions listed above, the contributions in detail are:

DL, CA, and VMI were responsible for strain construction and performance of *in vitro* experiments. MRC, PC, and MJJ designed and performed *in vitro* GI simulations. LR performed mouse experiments and analyzed all *in vivo* experiments. NGH and MRC conceived of and designed the *in silico* simulations and wrote the model code. MJJ developed and performed LC‐MS/MS analyses for all *in vitro* and *in vivo* studies. CGB and RBS were responsible for growing and lyophilizing cells for IVS and *in vivo* studies. J‐RG assisted with strain characterization and analysis. CK, MP, VMI, MRC and DLH supervised the work. DL and NGH wrote the manuscript, and all authors contributed to revisions of paper drafts.

## Disclosure and competing interests statement

DL, NGH, MJJ, PC, LR, CGB, RBS, CA, JG, CK, MP, MRC, VMI, and DLH are employees of Synlogic, Inc.

## Supporting information



Expanded View Figures PDF

Table EV1

Table EV2

Dataset EV1

Dataset EV2

Dataset EV3

Dataset EV4

## Data Availability

Jupyter notebooks for the *in silico* simulation are available for download at Zenodo:
Horvath, Nicholas. (2022). An engineered bacteria lowers urinary oxalate in preclinical and *in silico* models of hyperoxaluria. Zenodo. https://doi.org/10.5281/zenodo.5911889
The complete genome sequence of SYNB8802 is available at NCBI under BioProject: PRJNA780099 (https://www.ncbi.nlm.nih.gov/bioproject/PRJNA780099), Accession number: CP087958.
