# Supplementary material for: An engineered bacterial therapeutic lowers urinary oxalate in preclinical models and in silico simulations of enteric hyperoxaluria
Source: Mol Syst Biol. 2022 Mar 7;18(3):e10539. doi: 10.15252/msb.202110539 (PMC8899768; doi:10.15252/msb.202110539)
Supplement: Supplementary file 1 — Expanded View Figures PDF [file MSB-18-e10539-s001.pdf]

Expanded View Figures

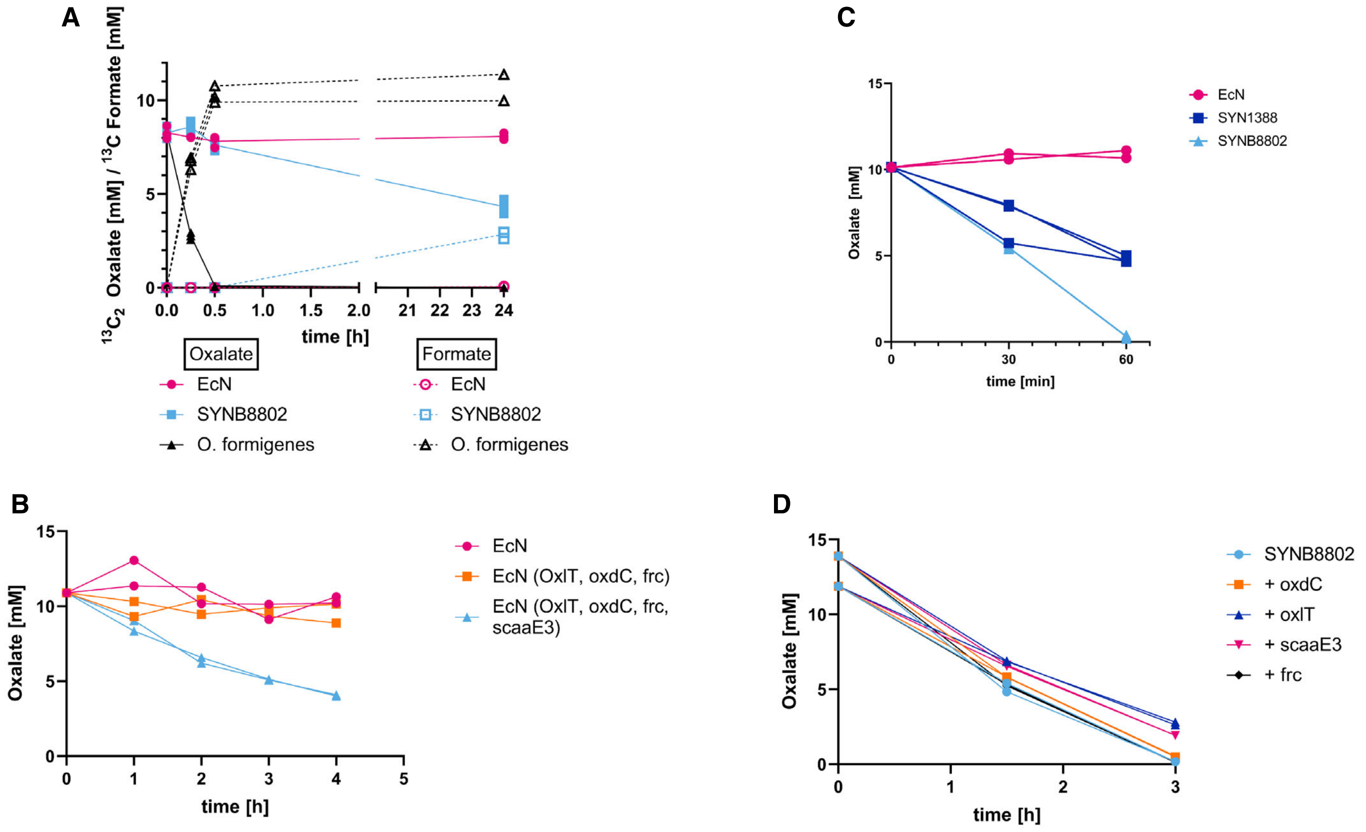

Figure EV1. SYNB8802 *in vitro* evaluation.

A SYNB8802 vs. *Oxalobacter formigenes*. Left *y*-axis: $^{13}C_2$-oxalate in mM, ^{13}C-formate in mM, *x*-axis: time in hours. *Oxalobacter formigenes* (black, triangle, solid line: oxalate, dotted line: formate). SYNB8802 (light blue, square, solid line: oxalate, dotted line: formate). The control EcN (pink, circle, solid line: oxalate, dotted line: formate). Biological triplicates were run and plotted separately.

B Scaae3 is needed for oxalate degradation in EcN. *Y*-axis shows oxalate in mM. *X*-axis: time in hours. Wild type EcN (pink, circle). EcN expressing the OF genes *oxdC, oxlT*, and *frc* on p15a (orange, square). EcN expressing the OF genes including *scaaE3* on p15a (light blue, triangle). Biological duplicates were run and plotted separately.

C SYNB8802 vs. SYN1388. *Y*-axis shows oxalate in mM. *X*-axis: time in minutes. Wild type EcN (pink, circle). Prototype strain SYN1388 (blue, square). SYNB8802 (light blue, triangle). Biological duplicates were run and plotted separately.

D Overexpression of individual pathway components. *Y*-axis shows oxalate in mM. *X*-axis: time in hours. SYNB8802 (blue, circle). SYNB8802, with added plasmid-based expression of *oxdC* (orange, square). SYNB8802, with added plasmid-based expression of *oxlT* (blue, triangle). SYNB8802, with added plasmid-based expression of *scaaE3* (pink, triangle). SYNB8802, with added plasmid-based expression of *frc* (black, diamond). Biological duplicates were run and plotted separately.

 © 2022 Synlogic

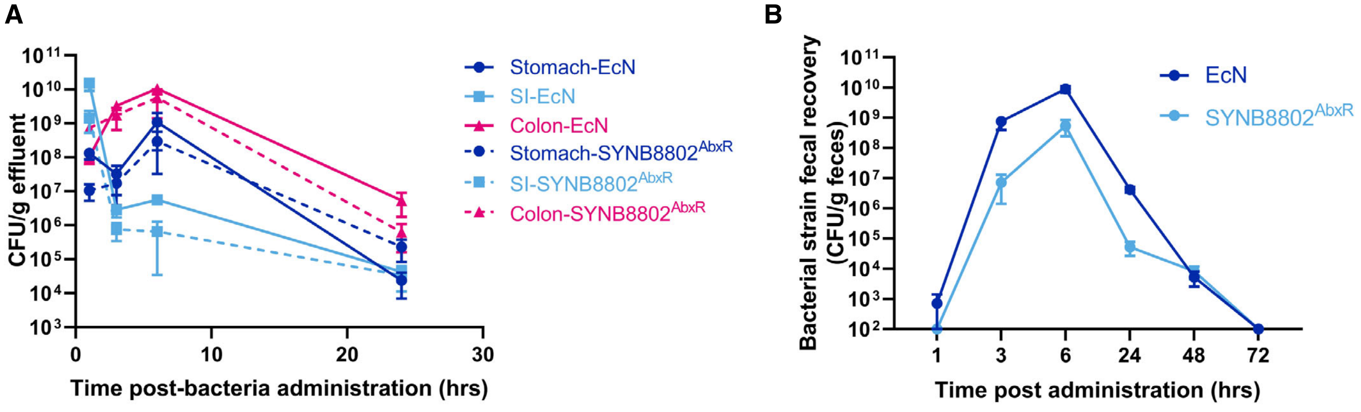

Figure EV2. EcN and SYNB8802 intestinal distribution and fecal clearance in mice.

A EcN and SYNB8802^{AbxR} *in vivo* kinetics. Abundance of EcN or SYNB8802^{AbxR} in gut effluents of healthy mice following single oral dose of bacteria. Data represented as mean ± SEM, $n = 4$/group/timepoint. Full lines represent EcN and hatched lines represent SYNB8802^{AbxR}.

B EcN and SYNB8802^{AbxR} fecal clearance. Abundance of EcN or SYNB8802^{AbxR} in fecal pellets of healthy mice following single oral dose of bacteria. Data represented as mean ± SEM, $n = 5$/group. Dark blue curve represents EcN and light blue curve represent SYNB8802^{AbxR}.

© 2022 Synlogic

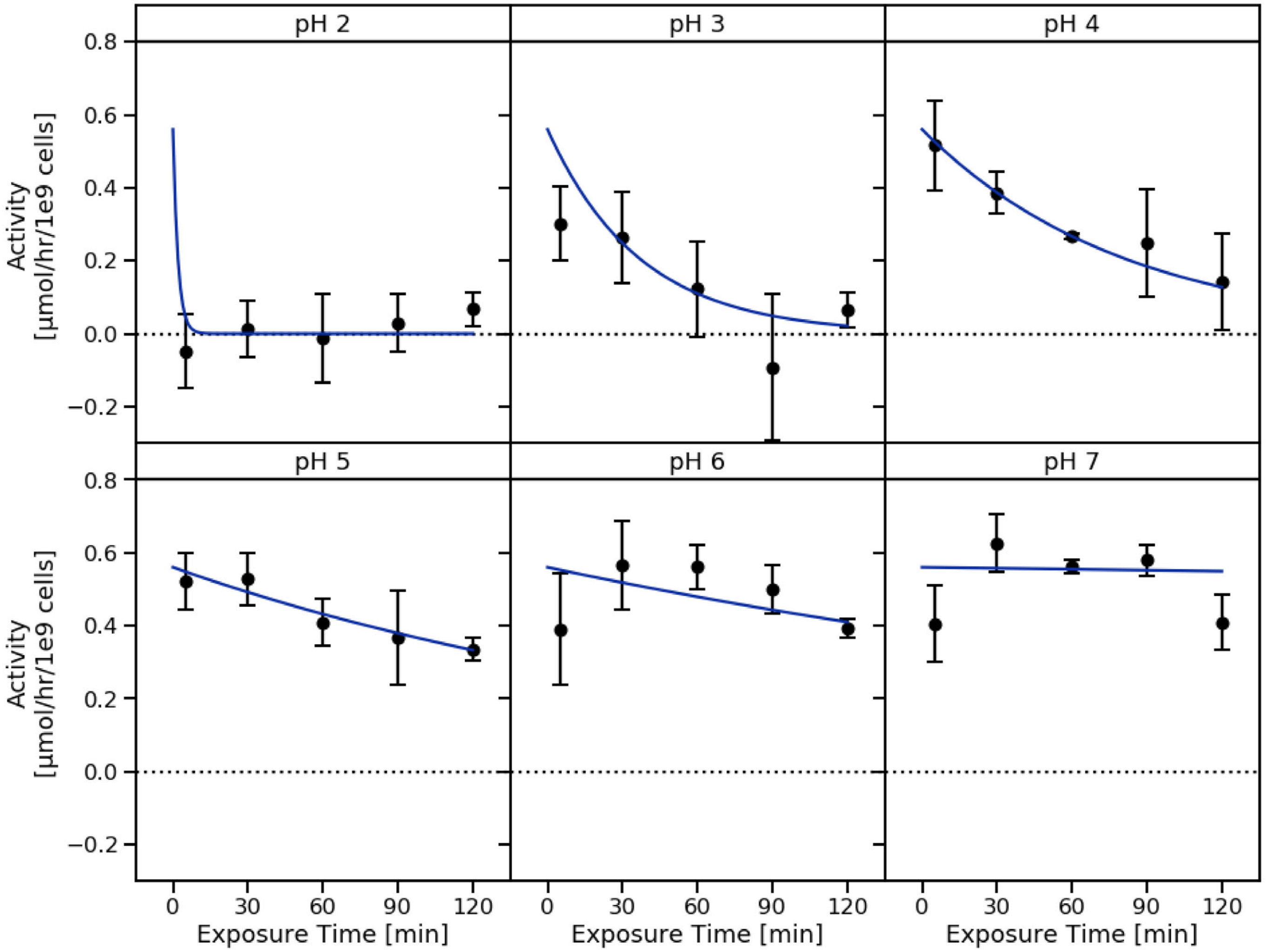

Figure EV3. SYNB8802 pH inhibition *in vitro* simulation.

SYNB8802 activity as a function of exposure time to medium at pH ranging from 2.0 to 7.0. Points and error bars in black represent *in vitro* measurements ($n = 3$ replicate cultures per group; mean ± SD). Blue curves represent exponential decay models fit to in vitro measurements for each pH level.

 © 2022 Synlogic

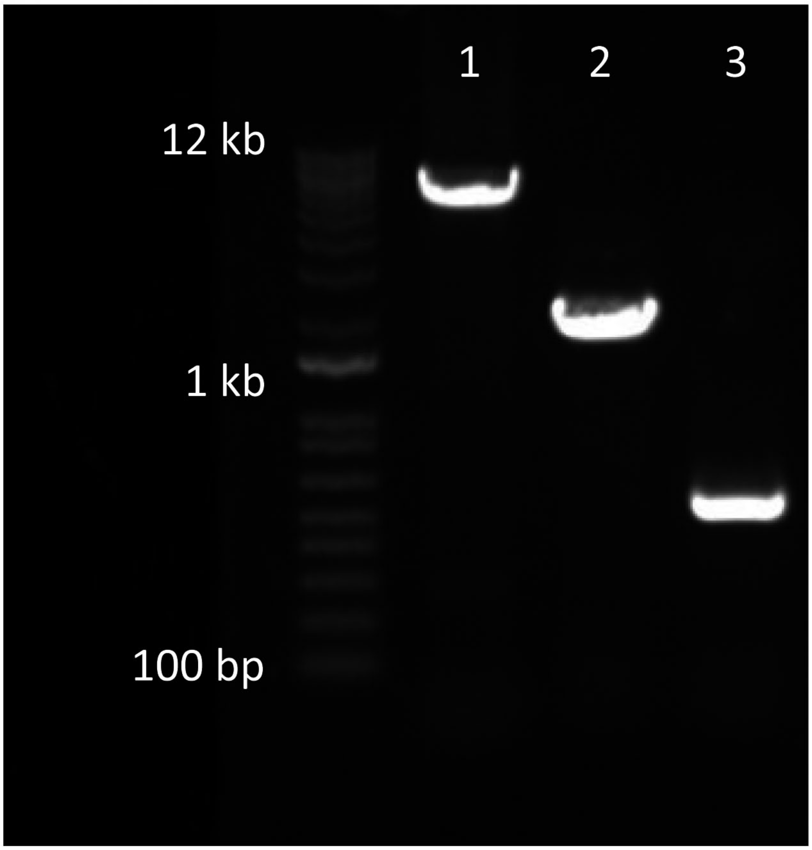

Figure EV4. Agarose gel of PCR verification for SYNB8802.

Lane 1 shows insertion of *agaI/rsmI*:: P_{FnrS} -*scaaE3-oxdC-frc*. Lane 2 shows insertion of *exo/cea*::P_{FnrS}-*oxlT*. Lane 3 shows knock out *thyA*.
