# Supplementary material for: An engineered bacterial therapeutic lowers urinary oxalate in preclinical models and in silico simulations of enteric hyperoxaluria
Source: Mol Syst Biol. 2022 Mar 7;18(3):e10539. doi: 10.15252/msb.202110539 (PMC8899768; doi:10.15252/msb.202110539)
Supplement: Supplementary file 8 [file MSB-18-e10539-s002.pdf]

molecular systems biology

An engineered bacterial therapeutic lowers urinary oxalate in preclinical models and in silico simulations of enteric hyperoxaluria

David Lubkowicz, Nicholas Horvath, Michael James, Pasquale Cantarella, Lauren Renaud, Christopher Bergeron, Ron Shmueli, Cami Anderson, Jian-Rong Gao, Caroline B. Kurtz, Mylene Perreault, Mark Charbonneau,, Vincent Isabella, and David Hava
DOI: 10.15252/msb.202110539

Corresponding author(s): David Lubkowicz (david@synlogictx.com)

Review Timeline:

Submission Date:	29th Jun 21
Editorial Decision:	13th Aug 21
Revision Received:	26th Nov 21
Editorial Decision:	7th Jan 22
Revision Received:	28th Jan 22
Accepted:	31st Jan 22

Editor: Maria Polychronidou

Transaction Report:

(Note: With the exception of the correction of typographical or spelling errors that could be a source of ambiguity, letters and reports are not edited. Depending on transfer agreements, referee reports obtained elsewhere may or may not be included in this compilation. Referee reports are anonymous unless the Referee chooses to sign their reports.)

1st Editorial Decision 13th Aug 2021

Thank you again for submitting your work to Molecular Systems Biology. We have now heard back from the three referees who agreed to evaluate your study. Overall, the reviewers acknowledge that the study is relevant. They raise however a series of concerns, which we would ask you to address in a major revision.

Without repeating all the points listed below, some of the more fundamental issues are the following:

- A more detailed and transparent documentation of results, strains, experimental procedures, animal experiments, strain optimization process etc. is required.
- The efficacy of the engineered strain needs to be better supported e.g. by performing direct comparisons.
- The reviewers mention that additional analyses are required to better support the utility of the presented model.
- The reviewers make recommendations for analyses to better support several of the reported conclusions and enhance the impact of the study.

All issues raised by the reviewers need to be satisfactorily addressed. Please contact me in case you would like to discuss in further detail any of the issues raised.

On a more editorial level, we would ask you to address the following points:

REFEREE REPORTS

Reviewer #1:

Lubkowicz et. al describe the design and implementation of a therapeutic strain of Escherichia coli to treat enteric hyperoxaluria (EH). As with some of their previous work, they use the well-characterized probiotic strain Nissle 1917 to convert toxic metabolites in the gut to prevent their accumulation and therefore disease. Using a pathway derived from a natural oxalate fermenter, Oxalobacter formigenes, supplemented with a yeast enzyme, the authors engineered the strain to metabolize oxalate to formate and CO_2 in vitro and in mouse and NHP models. Further, the authors construct a comprehensive in silico model to evaluate the effects of diet on urinary oxalate secretion and how inclusion of their therapeutic strain may lower urinary oxalate in healthy or diseased subjects. Overall, the group describes a compelling approach to treat EH using engineered bacteria and establish a future framework that will aid its evaluation in human subjects. Despite these admirable efforts, the manuscript could be improved with additional description of strain design and optimization as well as more transparent and thorough reporting of the animal data. The minimum data was included in this manuscript, which renders it difficult to ascertain the relevance of the reported work.
Specific Points:

- Throughout: While the advantages of an engineered, easy-to-manufacture, and well-characterized E. coli chassis are well described, the efficacy of the engineered strain is never compared to Oxalobacter formigenes, even in vitro. How does the rate of oxalate consumption compare between the engineered EcN strain and OF?
- Line 94-102: After the proof-of-concept strain is constructed, the authors embark on what seems like an extensive effort to render the strain suitable for therapeutic use. Briefly, the authors outline chromosomal integration, RBS optimization, promoter swapping etc. as part of these efforts. However, no data or extensive description is provided for these beyond the cursory description in the main text. For the benefit of others in the field interested in similar strain construction, can the data or a detailed description of this road to optimization be included in the supplement? At the very least, a comparison of the original and optimized versions of the strain?
- Line 103-105: Since CO_2 is a product of the decarboxylation reaction, does strain activity lead to acidification of the gut?
- Line 121-126: A bit more description and characterization of the in vivo model would be appreciated. Based on the methods, mice were gavaged with 100ug of labeled oxalate. How does this compare with the dietary levels consumed by patients with EH? Is the rate of oxalate consumption sufficient to treat a disease model? How does the metabolic activity of SYN8802 affect the endogenous murine microbiome in mice? I assume the CO_2 and formate produced by these strains may have an affect on the surrounding microbiota.
- Line 121-126/Fig 2a: In the methods, the authors describe that 60 mice were split into groups of n=15. However, the data from only 5 mice are depicted on the graph. Where is the data from the remaining 10 mice? Were these excluded from analysis? Were these mouse experiments reproducible? Why were only male mice used in the experiment?
- Line 127-137: The design/data analysis of the NHP experiments is confusing and slightly concerning. For both 2c and 2d, the variance of the vehicle control is very large, likely due to the aggregation of 4 separate days worth of experiments where different monkeys had different baseline levels of consumption/metabolism of the solution. How do cage effects and the fact that experiments were performed on different days manifest in the urinary oxalate levels? For the y-axis, is the 100% mark the mean urinary oxalate level to which all of the other samples are compared? For the sample groups, were the measurements compared to the aggregate mean of the vehicle control or the mean for that day of experiments? Were the experiments reproducible or performed a single time? Based on the study design in the Methods section, it seems like the sample groups were only performed a single time, whereas the vehicle control was performed 4 times. Given the variance of the vehicle group, it is difficult to discern if the effect is driven by the therapeutic strain or a cage/experiment effect. Finally, why are 2c and 2d represented as a fold change to a vehicle control whereas 2a and 2b are the raw oxalate values? Is the significance observed when looking at raw values or is the data transform necessary to observe the reduction?
- ISS Model: The proposed model seems like a useful tool to inform dosing. However, in the manuscript, it is used to demonstrate hypothetical efficacy of the therapeutic strain. Of course, the strain will perform as expected if the model is parameterized to include a consuming strain, provided the oxalate-metabolizing effect is not negligible. The real question is whether or not the strain can perform in humans given the altered biochemical state that may arise due to interpersonal variability or a diseased gut. In that case, the in vitro measured parameters may be skewed, such that the strain loses therapeutic value. Can the authors validate the engineered strains using a similar model parametrized to the murine or NHP models? In that case, the current in vivo data can be leveraged to validate the utility of the ISS model for future human work. Further, a slightly extended discussion about the necessity and utility of the ISS model would be appreciated.

Reviewer #2:

**Summary
*Describe your understanding of the story
The authors developed an engineered E. coli strain able to degrade oxalate from the environment into formate, with a plausible application for enteric hyperoxaluria treatment. Applying the proper biocontainment measures, they tested the strain activity in vitro with cultures in gastrointestinal-like medias and in vivo using mouse and non-human primate (NHP) EH models. After verifying the activity of the strain through this analysis, the authors developed an in silico mathematical model using literature parameters together with parameters derived from their in vitro studies. The model reflects the strain activity, its dynamics through the gastrointestinal tract and the dynamics of the oxalate transport and absorption. Finally, the authors compared the model to literature data in order to validate they could simulate an increased oxalate absorption and described the simulated implications of their strain if used in the clinics.

*What are the key conclusions: specific findings and concepts
-The authors created an engineered strain able to degrade oxalate in vitro and in vivo (rodents+NHPs).
-A computational model describes the potential benefits of using the oxalate degrading strain in humans

*What were the methodology and model system used in this study
-The model bacterial strain used is an engineered E. coli Nissle and it was tested in acute murine models (Acute 13C2-induced hyperoxaluria in C57BL/6 mice) and non-human primates (Non-naïve male cynomolgus monkeys). EH was induced using oxalate-rich diets. The methodology also includes common genetic engineering techniques (Gibson assembly...) and the strain engineering was performed with the common lambda red system. Other techniques used include LC/MS-MS for detection of radiolabeled compounds and general handling of animal models.
The mathematical framework developed seems to follow reasonable assumptions. The proposed ODE approach seems reasonable.

**General remarks
*Are you convinced of the key conclusions?
I am convinced the generated strain is able to degrade oxalate in vitro and in animal models, although the latter required the highest dose tested to be of significance. Regarding the mathematical model, the methodology used seems reasonable. However, I would be further convinced of the model's utility if it was further validated by adjusting the parameters for the animal models tested and see if they reproduce the experimental results.

*Place the work in its context.
Living therapeutics are a recent approach to treat diseases that classical drugs have not fully addressed. Our incremental knowledge regarding the gut microbiome is steadily enabling the use of probiotic strains not only to treat diseases but also to promote health among individuals. This knowledge combined with the engineering principles widely used in synthetic biology opens the door to the use of engineered bacteria as probiotics. This work serves as a great example of how the use of engineered probiotics can be of help in the case of enteric hyperoxaluria, a disease without an approved treatment up to date. I would also like to remark that Synlogic is a clear leader in this field as demonstrated by this and other pioneering efforts.

*What is the nature of the advance (conceptual, technical, clinical)?
The advance of this work is mainly translational. The concept of using engineered bacteria in the gut has been demonstrated before and the technical methods used are standard techniques in the field. However, the strain described in this work represents a potential advancement in the treatment of enteric hyperoxaluria and the technical experimental execution to validate it is outstanding.
Additionally, in my opinion the mathematical framework to assess the clinical potential of the strain has also some novelty. Although I would consider the mathematical approach rather a commonly used methodology, its application to predicting the outcome of EH treatment with engineering probiotics is original.

*How significant is the advance compared to previous knowledge?
The advance represents a solid step forward in the development of treatments for enteric hyperoxaluria. It is very remarkable the level of validation which includes NHPs. It is also another example of how engineered microbiome bacteria are becoming a validated approach to treat diseases where classical drugs have failed to meet the needs.

*What audience will be interested in this study?
I am sure researchers within the synthetic biology and microbiome fields will be interested in the study. Additionally, clinicians dealing with EH patients will be also very interested to see this study as a new treatment possibility as well as the patients themselves. I also think clinicians in general will be interested to follow another example of the development of engineered probiotics to fight diseases. I would also include the general public, as this study reflects how probiotics can become an established treatment option in the future.

**Major points
*Specific criticisms related to key conclusions
The model seems to compile reasonable assumptions towards the conclusion SYNB8802 degrades oxalate in vivo. However, I think it needs further validation to take further conclusions and demonstrate its real utility. Perhaps some supplementary figures showing the range of variability of the assumptions may help in clarifying the scope of the model.
Additionally, It would be highly desirable to remove all antibiotic resistance from SYNB8802. In the online methods section, it is mentioned how antibiotic resistances introduced during oxalate processing machinery introduction were removed (line 396). However, chloramphenicol resistance introduced to generate thyA auxotrophy seems to remain there despite being flanked by flippase (line 403). I was wondering whether this is an error or if the chloramphenicol resistance remains there.

*Specify experiments or analyses required to demonstrate the conclusions
For instance, doing an extreme values analysis would help to understand if despite the assumptions taken the theoretical effect of the strain is still valid. I think it would be interesting to simulate the range of oxalate elimination in function of different radial distributions of SYNB8802. Additionally, considering the simulations in a range of oxalate bioavailability assumptions might be beneficial to see the actual effect of SYNB8802.

*Motivate your critique with relevant citations and argumentation
In previous studies from the same authors demonstrated that the assumption of michaelis-menten like equations are valid for modeling Phe degradation in the gut using an engineered probiotic (Nelson, 2021). However, while in this previous study a comparison was made between in silico and in vivo data from NHP to further validate the model in this study there is only a comparison of oxalate absorption with previous clinical data. Although the assumptions taken will probably lead to an approximate effect of SYNB8802 using the ISS model, an extreme value analysis to establish some boundaries of the simulations within expected biological parameters could give stronger support to the computational model despite not being compared with experimental data.
M. T. Nelson, Characterization of an engineered live bacterial therapeutic for the treatment of phenylketonuria in a human gut-on-a-chip. Nature Communications. 12 (2021), doi:10.1038/s41467-021-23072-5.

**Minor points
*Easily addressable points
In line 89, It would help to explain what the hypothesis was based on to suspect oxalyl-CoA synthetase. Systematic analysis? Evidence from literature? Other experiments?
The strain SYN094 in line 494 is not described in the text nor the supplementary. Might it refer to wild EcN according to context?

*Presentation and style
No comments

*Trivial mistake
In line 68, "(17)." instead of ".(17)"
In line 105, "(Figure 1a)." instead of ".(Figure 1a)"
In line 223, "(37)." instead of ".(37)"
In line 121, SYN8802 should be SYNB8802 instead?
In line 274, the legend of figure 1b, I would suggest specifying which kind of line (continuous or slashed) belongs to which compound (oxalate or formate).

Reviewer #3:

Summary
The manuscript by Lubkowicz et al. develops and characterizes the effects of a novel engineered bacteria called SYNB8802 in lowering urinary oxalate. SYNB8802 is projected for therapeutic use in enteric hyperoxaluria (EH) that results in significant, irreversible, and progressive kidney damage.

The authors first describe the development of SYNB8802 an engineered bacteria designed to consume oxalate within the GI tract and convert it to nontoxic metabolites (formate and CO_2). SYNB8802 is genetically engineered using a non-colonizing strain of Escherichia coli Nissle 1917 (EcN) by engineering a pathway for oxalate degradation derived from the human commensal microorganism Oxalobacter formigenes into the probiotic strain EcN. The authors further engineer SYNB8802 as auxotrophic strain by deleting the thyA gene that encodes for thymidine synthetase, which is essential for replication, thus addressing biocontainment issues, an important clinical challenge in probiotic engineering.

The authors further demonstrate that oral administration of SYNB8802 leads to significantly decreased urinary oxalate excretion in healthy mice and non-human primates in a dose-dependent manner.

Additionally, simulation studies predict that administration of SYNB8802 can lead to clinically meaningful lowering of urinary

oxalate excretion in healthy volunteers and EH patients.

The authors conclude that SYNB8802 is a promising treatment for EH.

General remarks
While the work addresses an important metabolic disease and is novel, and provides conceptual, technical, and pre-clinical advancement to the field of probiotic bioengineering, I have multiple suggestions that I think would broaden the manuscript's impact and some areas that would require additional experiments to substantiate the authors' conclusions.

Major points
1. Fig. 1: Both PCR and WGS data confirming OxdC and associated effector components, OxLT pump, inducer-promoter pair (FNR promoter) and the thyA will be helpful and should be shared at least in the supplementary data.
2. Figure. 1c and supplementary figure 1 lacks the wild-type (WT) ECN control (as in Fig. 1b) which I believe is important in these figures as well.
3. In figure 2, a schematic of oral administration (dosing) and endpoints for both mouse and human primate models can be extremely helpful to increase readability.
4. While the authors use both the mice and human primate model (a major strength of the study) to investigate the effect of SYNB8802, in both model systems, the only parameter that was measured was urinary oxalate. I believe it is essential to take other parameters into account. What would happen to fecal oxalate and formate levels? Can urinary and fecal formate simultaneously with oxalate be measured (as in Fig. 1b)?
5. How well is SYNB8802 tolerated in in vivo models? Are there any serious or systemic adverse effects, relative to ECN wild-type controls? Are there any changes to gut barrier function (intestinal permeability, tight junctions, histopathology, mucus production, and goblet cells)?
6. Are there any changes to immune responses? A relative measurement (ECN wild-type and SYNB8802) using ELISA of major bio-immune markers in serum and/or harvested tissue samples will help address some of these concerns.
7. What is the microbial kinetics of SYNB8802? How long does SYNB8802 colonize in the gut? Although not evident, I believe the authors use an antibiotic marker (KanR) strain in these studies as the strain could be tracked. However, no such experiments were performed. An easy way to accomplish this would be to assess colonization by using a non-invasive fecal load measurements over time (Drolia, Rishi, et al. "Receptor-targeted engineered probiotics mitigate lethal Listeria infection." Nature communications 11.1 (2020): 1-23). How long does SYNB8802 survive outside?
8. How frequently the consumption of SYNB8802 is required in in vivo models to see the effects in lowering oxalate. Does this require continuous administration? Do the effects of SYNB8802 wane if not supplemented daily?
9. Figures 3-5 describe in vitro and in silico simulations and are informative. However, any phase 1 clinical data (which I believe the group has extensive expertise on) shared in this manuscript will substantially increase the impact and validate the predicted conclusions drawn in this study.

Minor Points.
1. Overall, the manuscript is well written. However, the discussion is a reiteration of the results section. It will be helpful to describe how probiotic bioengineering has combated other diseases (infectious and metabolic) using their own previous references (References 17, 20-23) and few references below and how their strategy represents a novelty.
a) Sola-Oladokun, B., Culligan, E. P. & Sleator, R. D. Engineered probiotics: applications and biological containment. Annu. Rev. Food Sci. Technol. 8, 353- 370 (2017).

b) Drolia, R. et al. Receptor-targeted engineered probiotics mitigate lethal Listeria infection. Nature communications 11, 1-23 (2020).

2. Line number 738-739 needs revision according to the journal-specific requirements.

Responses to reviewers

Reviewer 1

Question:

While the advantages of an engineered, easy-to-manufacture, and well-characterized E. coli chassis are well described, the efficacy of the engineered strain is never compared to Oxalobacter formigenes, even in vitro. How does the rate of oxalate consumption compare between the engineered EcN strain and OF?

Response from authors:

We have repeated the in vitro oxalate consumption and formate production experiment with *O. formigenes* as a control and included these results in Figure EV1d. The results are discussed in lines 95 - 97 of the result section and 284 - 292 of the discussion.

Question:

Line 94-102: After the proof-of-concept strain is constructed, the authors embark on what seems like an extensive effort to render the strain suitable for therapeutic use. Briefly, the authors outline chromosomal integration, RBS optimization, promoter swapping etc. as part of these efforts. However, no data or extensive description is provided for these beyond the cursory description in the main text. For the benefit of others in the field interested in similar strain construction, can the data or a detailed description of this road to optimization be included in the supplement? At the very least, a comparison of the original and optimized versions of the strain?

Response from authors:

To address these questions, we have added Figure panels EV1b and EV1c.1 b shows the original prototype strain, SYN1388 with plasmid based unoptimized expression compared to the final strain SYNBB8802. This is described in the main text in lines 119 - 120. In addition, we have included Figure EV1d, which shows that overexpression of the individual components on top the final engineered strain, SYNB8802, does not lead to increased activity. These results are also described in lines 123 - 126 in the result section.

Question:

Line 103-105: Since CO2 is a product of the decarboxylation reaction, does strain activity lead to acidification of the gut?

Response from authors:

While we have not directly measured the acidification of the gut, we believe that because we are using a genetic circuit naturally present in *O. formigenes*, which produces formate and CO_2 from oxalate in the human gut, that the risk of adverse events due to the metabolism of oxalate by SYNB8802 is low. Further, ~30% of the population is colonized with OF (Liu, 2017) and there are other microbiome members that can covert oxalate to formate and CO_2 (Hatch, 2017). To our knowledge, there are no described differences in GI pH or acidification in individuals colonized with OF compared to those who are uncolonized, and there are no significant adverse events reported in individuals colonized with OF.

Liu, M. et al. Oxalobacter formigenes-associated host features and microbial community structures examined using the American Gut Project. Microbiome 5(1), 108–125 (2017)
Hatch M. (2017). Gut microbiota and oxalate homeostasis. Annals of translational medicine, 5(2), 36. https://doi.org/10.21037/atm.2016.12.70

Question:

A bit more description and characterization of the in vivo model would be appreciated. Based on the methods, mice were gavaged with 100ug of labeled oxalate. How does this compare with the dietary levels consumed by patients with EH? Is the rate of oxalate consumption sufficient to treat a disease model? How does the metabolic activity of SYN8802 affect the endogenous murine microbiome in mice? I assume the CO2 and formate produced by these strains may have an effect on the surrounding microbiota.

Response from authors:

In this mouse pharmacology study, labeled oxalate was used as a tracer and the dose was selected based on tolerability and feasibility; the intent was not to directly mimic the dietary oxalate levels consumed by EH patients. However, studies have shown that healthy individuals consume approximately 200 mg of oxalate daily (Taylor et al. JASN 2007), or 2.9 mg/kg for a 70 kg individual. The amount of oxalate administered in our pharmacology studies was approximately 4 mg/kg, in the range of what a healthy individual would consume. The first sentence (above) was included in the method section of the manuscript to provide more description and rationale (lines 615-618).

Using an average in vitro rate of consumption of 1.25 µmol/hr*1e9 cells based on IVS studies (Figure 1c), the strain should be capable of consuming approximately 10 mg of oxalate, assuming 3 hours of consumption based on mouse gut transit. In a dietary mouse model induced with a low calcium high oxalate diet, and assuming 4 g of food ingested per day, mice

would ingest approximately 15 mg of oxalate on this diet. We thus expect that the rate of oxalate consumption should be sufficient to treat a dietary mouse model of hyperoxaluria. The authors agree that CO_2 and formate produced by SYNB8802 could have an impact on the mouse gut microbiome, but these studies have not been conducted. However, as discussed above, we don't expect our strain to produce more CO_2 than OF or other microbiome members would. In addition, the amount of CO_2 produced over time by the strain is not expected to influence the gut due to self-regulating gut homeostasis so the impact on gut microbiome, if any, should be minimal.

<u>Question:</u>

In the methods, the authors describe that 60 mice were split into groups of n=15. However, the data from only 5 mice are depicted on the graph. Where is the data from the remaining 10 mice? Were these excluded from analysis? Were these mouse experiments reproducible? Why were only male mice used in the experiment?

<u>Response from authors:</u>

We apologize for the confusion around number of animals and data points. A schematic of each study design has been added to Figure 2 for additional clarity. For the mouse experiment, 15 mice/group received the treatment, and mice were then grouped 3 per metabolic cage for urine collection, which resulted in 5 datapoints per group. The significant effects of SYNB8802^{AbxR} versus EcN were observed in 4 additional studies not reported here (a note was added in the methods). The studies were performed with male mice to avoid the potential confounding impact of the mouse estrous cycle and hormonal changes in our studies.

<u>Question:</u>

The design/data analysis of the NHP experiments is confusing and slightly concerning. For both 2c and 2d, the variance of the vehicle control is very large, likely due to the aggregation of 4 separate days worth of experiments where different monkeys had different baseline levels of consumption/metabolism of the solution. How do cage effects and the fact that experiments were performed on different days manifest in the urinary oxalate levels? For the y-axis, is the 100% mark the mean urinary oxalate level to which all of the other samples are compared? For the sample groups, were the measurements compared to the aggregate mean of the vehicle control or the mean for that day of experiments? Were the experiments reproducible or performed a single time? Based on the study design in the Methods section, it seems like the sample groups were only performed a single time, whereas the vehicle control was performed 4 times. Given the variance of the vehicle group, it is difficult to discern if the effect is driven by the therapeutic strain or a cage/experiment effect. Finally, why are 2c and 2d represented as a fold change to a vehicle control whereas 2a and 2b are the raw oxalate values? Is the significance observed when looking at raw values or is the data transform necessary to observe the reduction?

Response from authors:

The reviewer is correct that Figures 2c and 2d are an aggregation of 4 separate studies performed with the same set of 12 monkeys and each experiment, which included a vehicle group and a specific dose of SYNB8802^{AbxR}, was performed once. Statistical power analysis of the urinary oxalate data across multiple studies in this colony of monkeys suggested that about 2/3 of the variability is driven by inter-animal variability, suggesting minimal impact of day-to-day on urinary oxalate levels. Group assignment was alternated across studies and data was expressed as percent change from the respective vehicle for the day of the experiment to minimize the day-to-day impact on the experimental results. However, the interpretation is similar whether the data is expressed as absolute oxalate levels or percent change from vehicle, with a relatively flat dose response and a significant effect observed at $1e^{12}$ CFU. A few clarifying sentences were added to the method section describing this work (lines 697-701).

Question:

ISS Model: The proposed model seems like a useful tool to inform dosing. However, in the manuscript, it is used to demonstrate hypothetical efficacy of the therapeutic strain. Of course, the strain will perform as expected if the model is parameterized to include a consuming strain, provided the oxalate-metabolizing effect is not negligible. The real question is whether or not the strain can perform in humans given the altered biochemical state that may arise due to interpersonal variability or a diseased gut. In that case, the in vitro measured parameters may be skewed, such that the strain loses therapeutic value. Can the authors validate the engineered strains using a similar model parametrized to the murine or NHP models? In that case, the current in vivo data can be leveraged to validate the utility of the ISS model for future human work. Further, a slightly extended discussion about the necessity and utility of the ISS model would be appreciated.

Response from authors:

We agree that the modeling framework presented herein does not perfectly simulate the physiology of enteric hyperoxaluria. However, we believe that the assumptions surrounding the most relevant aspects of the gut microenvironment, namely pH, oxygen, and oxalate abundance, represent the typical EH patient, with interpersonal variability captured through the uncertainty in the fundamental aspect of the disease state, oxalate absorption. Further, the impact of these variables on strain action has been investigated. For example, considering not just the immediate effect of acidic pH but its propensity to reduce strain activity chronically. Regarding model validation, the reviewer is correct that recapitulation of strain efficacy in murine or NHP models would strengthen the predictions of the human-parametrized framework. However, we believe the current dataset is insufficient to achieve this validation, specifically that urinary oxalate has only been measured to 6 hours post-dosing. Due to the importance of the colon to oxalate absorption and strain action, model validation would require oxalate measurements over a significantly longer time course. The approach does, however, leverage clinical data to validate the connection between dietary oxalate and the urinary

endpoint. Further, at lines 74 - 80 the manuscript points to a previous demonstration of the success of the approach, wherein an *in silico* model of phenylketonuria, mechanistically analogous to the one presented here, successfully predicted strain function in non-human primates and healthy subjects. The discussion of the utility of mathematical modeling has been extended at lines 74 - 80 as per the reviewer's suggestion.

Reviewer 2

Question:

The model seems to compile reasonable assumptions towards the conclusion SYNB8802 degrades oxalate in vivo. However, I think it needs further validation to take further conclusions and demonstrate its real utility. Perhaps some supplementary figures showing the range of variability of the assumptions may help in clarifying the scope of the model.

Response from authors:

We appreciate the reviewer's point about the range of variability of the model assumptions. Please see the comment below regarding the extreme values analysis that has been added to the manuscript.

Question:

Additionally, It would be highly desirable to remove all antibiotic resistance from SYNB8802. In the online methods section, it is mentioned how antibiotic resistances introduced during oxalate processing machinery introduction were removed (line 396). However, chloramphenicol resistance introduced to generate thyA auxotrophy seems to remain there despite being flanked by flippase (line 403). I was wondering whether this is an error or if the chloramphenicol resistance remains there.

Response from authors:

This is an error, SYNB8802 does not contain an antibiotic resistance gene and is clarified in lines 556 – 558. The use of the antibiotic resistant strains was specific to use in the animal studies and is not a feature of the ultimate clinical candidate.

Question:

For instance, doing an extreme values analysis would help to understand if despite the assumptions taken the theoretical effect of the strain is still valid. I think it would be interesting to simulate the range of oxalate elimination in function of different radial distributions of SYNB8802. Additionally, considering the simulations in a range of oxalate bioavailability assumptions might be beneficial to see the actual effect of SYNB8802

Response from authors:

The authors appreciate and agree with the reviewer's suggestion to perform an extreme values analysis and have added this to the manuscript (Dataset EV4). The model prediction of urinary oxalate reduction due to SYNB8802 is generally robust to alternate assumptions, with the greatest discrepancies resulting from strain activity parameters (maximal enzyme velocity and Michaelis constant) followed by plasma-associated parameters (endogenous production rate and urinary excretion rate constant). A discussion of this analysis has been added at lines 234 – 238.

While we agree that simulating radial distribution of SYNB8802 would be interesting, as well as impactful for secretion-based therapeutic approaches, we do not believe that it will have a meaningful effect on dietary removal in the gut. We assume that dietary oxalate and SYNB8802 enter the stomach together with meals and are well mixed, and that dietary removal of oxalate lessens oxalate absorbed into plasma regardless of radial distribution. The bioavailability of oxalate, however, is a key component of the modeling approach and is currently addressed through a global absorption fraction that captures variability in patient physiology.

Question:

In line 89, It would help to explain what the hypothesis was based on to suspect oxalyl-CoA synthetase. Systematic analysis? Evidence from literature? Other experiments?

The strain SYN094 in line 494 is not described in the text nor the supplementary. Might it refer to wild EcN according to context?

Response from authors:

Oxalobacter formigenes metabolizes oxalyl-CoA to formyl-CoA and CO_2. There are no pathways described in *E. coli* to generate either oxalyl-CoA or formyl-CoA from oxalate or formate respectively. A literature search on oxalate to oxalyl-CoA conversion identified the gene *scaaE3* which is utilized for oxalate metabolism *S. cerevisiae*. We were unable to find an enzyme to convert formate to formyl-CoA, which based on the cyclic nature of the pathway should also work to start the pathway. We synthesized a codon optimized version of *scaaE3* which enabled the oxalobacter derived pathway to work. A description of this was added in line 100 - 107

Correct, SYN094 refers to wild-type EcN and was updated as such in the main text.

Question:

In line 68, "(17)." instead of ".(17)" - CORRECTED

In line 105, "(Figure 1a)." instead of ".(Figure 1a)" - CORRECTED

In line 223, "(37)." instead of ".(37)" - CORRECTED

In line 121, SYN8802 should be SYNB8802 instead? - CORRECTED

In line 274, the legend of figure 1b, I would suggest specifying which kind of line (continuous or slashed) belongs to which compound (oxalate or formate). – CORRECTED

Reviewer 3

<u>Questions:</u>

Fig. 1: Both PCR and WGS data confirming OxdC and associated effector components, OxLT pump, inducer-promoter pair (FNR promoter) and the thyA will be helpful and should be shared at least in the supplementary data.

<u>Response from authors:</u>

Submitted WGS, BioProject: PRJNA780099, Accession number: CP087958
Added in the manuscript Lines 948 - 949
Agarose gel of PCR confirming inserts is provided in Figure EV4 and corresponding primers are found in Table EV2

<u>Questions:</u>

2. Figure. 1c and supplementary figure 1 lacks the wild-type (WT) ECN control (as in Fig. 1b) which I believe is important in these figures as well.

<u>Response from authors:</u>

We acknowledge the importance of including wild-type EcN as a control in all in vitro experiments and have added it to Figure 1b and Figure EV1a, EV1b and Ev1c.

Regarding Figure 1c:

We have tested wt EcN in various in vitro experiments and have not observed any oxalate degrading capabilities. The IVS is a complex and extensive experiment and due to the lack of oxalate degradation in wt EcN, it was not included in this specific study. The purpose of the IVS is specific to determining the impact of the various GI compartments (composition, pH, oxygen) on the activity of the final candidate strain SYNB8802. The simpler in vitro activity assay was used to compare engineered strains versus wild type, as can be seen in Figure 1b and Figure EV1a, EV1b and EV1c.

<u>Questions:</u>

While the authors use both the mice and human primate model (a major strength of the study) to investigate the effect of SYNB8802, in both model systems, the only parameter that was

measured was urinary oxalate. I believe it is essential to take other parameters into account. What would happen to fecal oxalate and formate levels? Can urinary and fecal formate simultaneously with oxalate be measured (as in Fig. 1b)?

Response from authors:
Urinary oxalate is the clinical endpoint and most relevant disease biomarker for this indication. The mechanism of action of SYNB8802 is to reduce urinary oxalate to prevent kidney damage. Fecal oxalate excretion is the normal route of dietary oxalate removal from the body. Therefore, we do not think that using fecal oxalate is highly informative. We have seen inconsistent changes in SYNB8802-driven fecal oxalate in 3 mouse experiments and no significant difference in fecal oxalate and $^{13}C_2$-oxalate recovery was detected at both 6- and 24-hours post dose in nonhuman primates.

Regarding the utilization of formate as a biomarker of strain activity, formate is ubiquitous, as it is a terminal metabolite of many microbial processes within the gut (Pietzke, 2020). Due to the natural abundance of formate in the normal gut, it has not been possible to detect the comparatively small amount of formate produced by SYNB8802 in any of the animal samples evaluated.

Pietzke M, Meiser J, Vazquez A. Formate metabolism in health and disease. Mol Metab. 2020 Mar;33:23-37. doi: 10.1016/j.molmet.2019.05.012.

Questions:

Are there any changes to immune responses? A relative measurement (ECN wild-type and SYNB8802) using ELISA of major bio-immune markers in serum and/or harvested tissue samples will help address some of these concerns.

Response from authors:

We have not specifically measured immune biomarkers in any of our samples considering the established record of safe EcN use for over 100 years in the treatment of various GI conditions, including inflammatory bowel disease and irritable bowel syndrome. In addition to a long-term safety profile, the microbiological, biochemical, and genetic attributes of EcN have been thoroughly characterized. EcN does not exhibit any virulence factors, is non-pathogenic, is killed upon exposure to serum, contains no antibiotic resistance determinants and is unable to stably colonize the human GI tract after long-term use. In fact, studies have shown that EcN can exert anti-inflammatory effects in LPS-induced sepsis in mice (Arribas, 2009) and similar positive effects have been reported in several mouse models of inflammatory bowel disease (Sonnenborn, 2009). In addition, studies have shown normal plasma levels of inflammatory cytokines, including IL-6 and TNFα, after multiple days of dosing despite observation of a small and reversible increase following the first dose in healthy animals (Lin, 2021).

Arribas B, Rodríguez-Cabezas ME, CamuescoD, Comalada M, Bailón E, Utrilla P, Nieto A, Concha A, Zarzuelo A, Gálvez J. A probiotic strain of *Escherichia coli*, Nissle 1917, given orally exerts local and systemic anti-inflammatory effects in lipopolysaccharide-induced sepsis in mice BJP 2009 157:1024-1033, doi:10.1111/j.1476-5381.2009.00270.x

Sonnenborn U, Schulze, J. The non-pathogenic *Escherichia coli* strain Nissle 1917 – features of a versatile probiotic. Microbial Ecology in Health and Disease 2009, 21:3-4, 122-158, DOI: 10.3109/08910600903444267

Lin S, Mukherjee S, Li J, Hou W, Pan C, Liu J. Mucosal immunity-mediated modulation of the gut microbiome by oral delivery of probiotics into Peyer's patches. *Sci. Adv.* 2021; 7 (20) : eabf0677 DOI: 10.1126/sciadv.abf0677

<u>Questions:</u>

In figure 2, a schematic of oral administration (dosing) and endpoints for both mouse and human primate models can be extremely helpful to increase readability.

<u>Response from authors:</u>

The schematics have been included in figure 2.

<u>Questions:</u>

How well is SYNB8802 tolerated in in vivo models? Are there any serious or systemic adverse effects, relative to ECN wild-type controls? Are there any changes to gut barrier function (intestinal permeability, tight junctions, histopathology, mucus production, and goblet cells)?

<u>Response from authors:</u>

The studies performed in support of this publication were done in animals that received a single dose of SYNB8802 in monkeys or 2 doses of SYNB8802 in mice. The treatment was well tolerated in both species and no serious or systemic adverse effects were observed. The changes to gut barrier functions were not evaluated in the studies performed to support this work considering the short exposure to SYNB8802 for these studies.

<u>Questions:</u>

How frequently the consumption of SYNB8802 is required in in vivo models to see the effects in lowering oxalate. Does this require continuous administration? Do the effects of SYNB8802 wane if not supplemented daily?

Response from authors:

In the studies presented, mice received 2 doses of the engineered probiotic and nonhuman primates received a single dose. It is expected that continuous administration will be required in humans due to the non-colonizing nature of EcN derived SYNB8802. The non-colonizing nature is described in the text in lines 67-73. From animal models and *in silico* simulation, we expect three times daily dosing with SYNB8802 to be necessary to maintain therapeutic efficacy at the level presented herein; efficacy is expected to wane after cessation of dosing. Further details around dosing regimen are expected to become clearer during clinical trials.

Questions:

What is the microbial kinetics of SYNB8802? How long does SYNB8802 colonize in the gut? Although not evident, I believe the authors use an antibiotic marker (KanR) strain in these studies as the strain could be tracked. However, no such experiments were performed. An easy way to accomplish this would be to assess colonization by using a non-invasive fecal load measurements over time (Drolia, Rishi, et al. "Receptor-targeted engineered probiotics mitigate lethal Listeria infection." Nature communications 11.1 (2020): 1-23). How long does SYNB8802 survive outside?

Response from authors:

Studies done in healthy mice with EcN and SYNB8802^{KanR} demonstrated very similar kinetics and excretion patterns between the strains. By 1 hour, both strains were most abundant in the small intestine and rapidly cleared so that by 24 hours, the majority of the cells are found in the colon. When measuring the excretion of EcN and SYNB8802^{KanR} in the feces, we observed that both strains were completely excreted by 72 hours, suggesting that the engineering of SYNB8802 did not impact the kinetics and excretion profile as compared to EcN. This data (Figure EV2) has been added to the revised version of the manuscript within the supplementary information.

Questions:

Figures 3-5 describe in vitro and in silico simulations and are informative. However, any phase 1 clinical data (which I believe the group has extensive expertise on) shared in this manuscript will substantially increase the impact and validate the predicted conclusions drawn in this study.

Response from authors:

Our Phase 1 study is still ongoing and is not ready at this point of time for publication. We are aiming to submit a separate clinical publication for our Phase 1a/b data when those data are available.

Minor Points from reviewer

1. Overall, the manuscript is well written. However, the discussion is a reiteration of the results section. It will be helpful to describe how probiotic bioengineering has combated other diseases (infectious and metabolic) using their own previous references (References 17, 20-23) and few references below and how their strategy represents a novelty.

a) Sola-Oladokun, B., Culligan, E. P. & Sleator, R. D. Engineered probiotics: applications and biological containment. Annu. Rev. Food Sci. Technol. 8, 353- 370 (2017).

b) Drolia, R. et al. Receptor-targeted engineered probiotics mitigate lethal Listeria infection. Nature communications 11, 1-23 (2020.

Response from authors:

Added onto discussion lines 250 - 266

2. Line number 738-739 needs revision according to the journal-specific requirements.

- **CORRECTED**

Thank you for sending us your revised manuscript. We have now heard back from the three reviewers who were asked to evaluate your revised study. As you will see below, the reviewers are satisfied with the modifications made and are overall supportive of publication. Reviewer #1 expresses some remaining reservations, which do not preclude the publication of the study, but could be addressed by including a couple of sentences in the discussion, referring to the potential effects of the living biotherapeutic on the gut microbiome etc.

Before we can proceed with formally accepting the study for publication, we would also ask you to address some minor editorial issues listed below.

REFEREE REPORTS

Reviewer #1:

The authors sufficiently addressed my previous concerns with the manuscript. The additional data and text have improved the quality of manuscript and its clarity. I recommend publication with minor and highly subjective reservations:
It is clear that the goal of the study was to develop a living biotherapeutic for future implementation in human studies. As a result, the preclinical endpoints are narrow and well-defined. What is lost in this approach is a more academic and thorough exploration of the experimental system to uncover how the activity of these strains may impact the microbiome or animal physiology and immunity in unintended ways. True, O. formicigenerens is a common gut microbe that naturally performs the engineered metabolic function and the risk of adverse events is low, but the activity of oxalate degradation may have impacts on the microbial community and the host. The experimental system developed herein is primed to perform these potentially illuminating studies, but the pragmatic focus of the study leaves a number of these potentially interesting avenues unexplored.

Reviewer #2:

Issues raised have been addressed adequately and the manuscript seems ready for publication

Reviewer #3:

Lubkowicz and colleagues have performed a significant amount of work to improve and revise the manuscript. I think the manuscript is impactful and of broad interest to the field. My comments are answered, and I have no further comments.

The authors have made all requested editorial changes.

ACCEPTED **31st Jan 2022**

Thank you again for sending us your revised manuscript. We are now satisfied with the modifications made and I am pleased to inform you that your paper has been accepted for publication.

EMBO PRESS

YOU MUST COMPLETE ALL CELLS WITH A PINK BACKGROUND ↓

PLEASE NOTE THAT THIS CHECKLIST WILL BE PUBLISHED ALONGSIDE YOUR PAPER

Corresponding Author Name: David Lubkowicz

Journal Submitted to: Mol Sys Bio

Manuscript Number: MSB-2021-10539R

USEFUL LINKS FOR COMPLETING THIS FORM

http://www.antibodypedia.com
http://1degreebio.org
http://www.equator-network.org/reporting-guidelines/improving-bioscience-research-repor
http://grants.nih.gov/grants/olaw/olaw.htm
http://www.mrc.ac.uk/Ourresearch/Ethicsresearchguidance/Useofanimals/index.htm
http://ClinicalTrials.gov
http://www.consort-statement.org
http://www.consort-statement.org/checklists/view/32-consort/66-title
http://www.equator-network.org/reporting-guidelines/reporting-recommendations-for-tumo
http://datadryad.org
http://figshare.com
http://www.ncbi.nlm.nih.gov/gap
http://www.ebi.ac.uk/ega
http://biomodels.net/
http://biomodels.net/miriam/
http://jjj.biochem.sun.ac.za
https://osp.od.nih.gov/biosafety-biosecurity-and-emerging-biotechnology/
http://www.selectagents.gov/

Reporting Checklist For Life Sciences Articles (Rev. June 2017)

This checklist is used to ensure good reporting standards and to improve the reproducibility of published results. These guidelines are consistent with the Principles and Guidelines for Reporting Preclinical Research issued by the NIH in 2014. Please follow the journal's authorship guidelines in preparing your manuscript.

A- Figures
1. Data
The data shown in figures should satisfy the following conditions:

- ➔ the data were obtained and processed according to the field's best practice and are presented to reflect the results of the experiments in an accurate and unbiased manner.
- ➔ figure panels include only data points, measurements or observations that can be compared to each other in a scientifically meaningful way.
- ➔ graphs include clearly labeled error bars for independent experiments and sample sizes. Unless justified, error bars should not be shown for technical replicates.
- ➔ if n< 5, the individual data points from each experiment should be plotted and any statistical test employed should be justified
- ➔ Source Data should be included to report the data underlying graphs. Please follow the guidelines set out in the author ship guidelines on Data Presentation.

2. Captions
Each figure caption should contain the following information, for each panel where they are relevant:

- ➔ a specification of the experimental system investigated (eg cell line, species name).
- ➔ the assay(s) and method(s) used to carry out the reported observations and measurements
- ➔ an explicit mention of the biological and chemical entity(ies) that are being measured.
- ➔ an explicit mention of the biological and chemical entity(ies) that are altered/varied/perturbed in a controlled manner.

- ➔ the exact sample size (n) for each experimental group/condition, given as a number, not a range;
- ➔ a description of the sample collection allowing the reader to understand whether the samples represent technical or biological replicates (including how many animals, litters, cultures, etc.).
- ➔ a statement of how many times the experiment shown was independently replicated in the laboratory.
- ➔ definitions of statistical methods and measures:
  - common tests, such as t-test (please specify whether paired vs. unpaired), simple χ2 tests, Wilcoxon and Mann-Whitney tests, can be unambiguously identified by name only, but more complex techniques should be described in the methods section;
  - are tests one-sided or two-sided?
  - are there adjustments for multiple comparisons?
  - exact statistical test results, e.g., P values = x but not P values < x;
  - definition of 'center values' as median or average;
  - definition of error bars as s.d. or s.e.m.

Any descriptions too long for the figure legend should be included in the methods section and/or with the source data.

In the pink boxes below, please ensure that the answers to the following questions are reported in the manuscript itself. Every question should be answered. If the question is not relevant to your research, please write NA (non applicable). We encourage you to include a specific subsection in the methods section for statistics, reagents, animal models and human subjects.

B- Statistics and general methods

Please fill out these boxes ↓ (Do not worry if you cannot see all your text once you press return)

1.a. How was the sample size chosen to ensure adequate power to detect a pre-specified effect size?	For in vitro experiments: Assays have been performed in triplicate if not otherwise stated
1.b. For animal studies, include a statement about sample size estimate even if no statistical methods were used.	No sample size calculations were performed. The number of mice per group included in the study was based on similar experiments previously executed for other programs. The number of monkeys per study was determined by the number of animals available in the colony. These animals have previously been used for similar experiments with the same n per group.
2. Describe inclusion/exclusion criteria if samples or animals were excluded from the analysis. Were the criteria pre-established?	No samples were excluded
3. Were any steps taken to minimize the effects of subjective bias when allocating animals/samples to treatment (e.g. randomization procedure)? If yes, please describe.	No
For animal studies, include a statement about randomization even if no randomization was used.	For mouse studies, animals were assigned to groups based on body weight. For nonhuman primate studies, the first 6 animals were assigned to group 1 and the remaining 6 animals were assigned to group 2. Group assignments were alternated across studies.
4.a. Were any steps taken to minimize the effects of subjective bias during group allocation or/and when assessing results (e.g. blinding of the investigator)? If yes please describe.	No
4.b. For animal studies, include a statement about blinding even if no blinding was done	Mouse and nonhuman primate studies were done in an unblinded fashion.
5. For every figure, are statistical tests justified as appropriate?	Yes
Do the data meet the assumptions of the tests (e.g., normal distribution)? Describe any methods used to assess it.	Sample size is too small to assess normality
Is there an estimate of variation within each group of data?	Data is presented as mean +/- SEM.

Is the variance similar between the groups that are being statistically compared?	Variance was not statistically tested

C- Reagents

6. To show that antibodies were profiled for use in the system under study (assay and species), provide a citation, catalog number and/or clone number, supplementary information or reference to an antibody validation profile. e.g., Antibodypedia (see link list at top right), 1DegreeBio (see link list at top right).	No antibodies were used in these studies
7. Identify the source of cell lines and report if they were recently authenticated (e.g., by STR profiling) and tested for mycoplasma contamination.	No cell lines were used in these studies

* for all hyperlinks, please see the table at the top right of the document

D- Animal Models

8. Report species, strain, gender, age of animals and genetic modification status where applicable. Please detail housing and husbandry conditions and the source of animals.	Details included in manuscript
9. For experiments involving live vertebrates, include a statement of compliance with ethical regulations and identify the committee(s) approving the experiments.	Ethical statement included in the manuscript
10. We recommend consulting the ARRIVE guidelines (see link list at top right) (PLoS Biol. 8(6), e1000412, 2010) to ensure that other relevant aspects of animal studies are adequately reported. See author guidelines, under 'Reporting Guidelines'. See also: NIH (see link list at top right) and MRC (see link list at top right) recommendations. Please confirm compliance.	ARRIVE guidelines were consulted and animal studies are adequately reported.

E- Human Subjects

11. Identify the committee(s) approving the study protocol.	N/A
12. Include a statement confirming that informed consent was obtained from all subjects and that the experiments conformed to the principles set out in the WMA Declaration of Helsinki and the Department of Health and Human Services Belmont Report.	N/A
13. For publication of patient photos, include a statement confirming that consent to publish was obtained.	N/A
14. Report any restrictions on the availability (and/or on the use) of human data or samples.	N/A
15. Report the clinical trial registration number (at ClinicalTrials.gov or equivalent), where applicable.	N/A
16. For phase II and III randomized controlled trials, please refer to the CONSORT flow diagram (see link list at top right) and submit the CONSORT checklist (see link list at top right) with your submission. See author guidelines, under 'Reporting Guidelines'. Please confirm you have submitted this list.	N/A
17. For tumor marker prognostic studies, we recommend that you follow the REMARK reporting guidelines (see link list at top right). See author guidelines, under 'Reporting Guidelines'. Please confirm you have followed these guidelines.	N/A

F- Data Accessibility

18: Provide a "Data Availability" section at the end of the Materials & Methods, listing the accession codes for data generated in this study and deposited in a public database (e.g. RNA-Seq data: Gene Expression Omnibus GSE39462, Proteomics data: PRIDE PXD000208 etc.) Please refer to our author guidelines for 'Data Deposition'.<br><br>Data deposition in a public repository is mandatory for:<br>a. Protein, DNA and RNA sequences<br>b. Macromolecular structures<br>c. Crystallographic data for small molecules<br>d. Functional genomics data<br>e. Proteomics and molecular interactions	SYN8802 genome sequence is publically available, BioProject: PRJNA780099, Accession number: CP087958
19. Deposition is strongly recommended for any datasets that are central and integral to the study; please consider the journal's data policy. If no structured public repository exists for a given data type, we encourage the provision of datasets in the manuscript as a Supplementary Document (see author guidelines under 'Expanded View' or in unstructured repositories such as Dryad (see link list at top right) or Figshare (see link list at top right).	Raw source data for all figures can be requested. All other datasets of importance are included in Expanded View
20. Access to human clinical and genomic datasets should be provided with as few restrictions as possible while respecting ethical obligations to the patients and relevant medical and legal issues. If practically possible and compatible with the individual consent agreement used in the study, such data should be deposited in one of the major public access-controlled repositories such as dbGAP (see link list at top right) or EGA (see link list at top right).	N/A
21. Computational models that are central and integral to a study should be shared without restrictions and provided in a machine-readable form. The relevant accession numbers or links should be provided. When possible, standardized format (SBML, CellML) should be used instead of scripts (e.g. MATLAB). Authors are strongly encouraged to follow the MIRIAM guidelines (see link list at top right) and deposit their model in a public database such as Biomodels (see link list at top right) or JWS Online (see link list at top right). If computer source code is provided with the paper, it should be deposited in a public repository or included in supplementary information.	Jupyter notebooks for the in silico simulation are available for download on zenodo.org

G- Dual use research of concern

22. Could your study fall under dual use research restrictions? Please check biosecurity documents (see link list at top right) and list of select agents and toxins (APHIS/CDC) (see link list at top right). According to our biosecurity guidelines, provide a statement only if it could.	N/A
